# User experience of home-based AbC-19 SARS-CoV-2 antibody rapid lateral flow immunoassay test

**DOI:** 10.1038/s41598-022-05097-y

**Published:** 2022-01-21

**Authors:** Min Jing, Raymond Bond, Louise J. Robertson, Julie Moore, Amanda Kowalczyk, Ruth Price, William Burns, M. Andrew Nesbit, James McLaughlin, Tara Moore

**Affiliations:** 1 Nanotechnology and Integrated Bioengineering Centre (NIBEC), School of Engineering, Ulster University, Jordanstown, UK; 2 School of Computing, Ulster University, Jordanstown, UK; 3 Biomedical Sciences Research Institute, Ulster University, Coleraine, UK; 4 Avellino USA, 1505 Adams Drive, Menlo Park, CA 94025 USA

**Keywords:** Public health, Computer science, Statistics

## Abstract

The urgent need to scale up testing capacity during the COVID-19 pandemic has prompted the rapid development of point-of-care diagnostic tools such as lateral flow immunoassays (LFIA) for large-scale community-based rapid testing. However, studies of how the general public perform when using LFIA tests in different environmental settings are scarce. This user experience (UX) study of 264 participants in Northern Ireland aimed to gather a better understanding of how self-administered LFIA tests were performed by the general public at home. The UX performance was assessed via analysis of a post-test questionnaire including 30 polar questions and 11 7-point Likert scale questions, which covers the multidimensional aspects of UX in terms of ease of use, effectiveness, efficiency, accuracy and satisfaction. Results show that 96.6% of participants completed the test with an overall average UX score of 95.27% [95% confidence interval (CI) 92.71–97.83%], which suggests a good degree of user experience and effectiveness. Efficiency was assessed based on the use of physical resources and human support received, together with the mental effort of self-administering the test measured via NASA Task Load Index (TLX). The results for six TLX subscales show that the participants scored the test highest for mental demand and lowest for physical demand, but the average TLX score suggests that the general public have a relatively low level of mental workload when using LFIA self-testing at home. Five printed LFIA testing results (i.e. the ‘simulated’ results) were used as the ground truth to assess the participant’s performance in interpreting the test results. The overall agreement (accuracy) was 80.63% [95% CI 75.21–86.05%] with a Kappa score 0.67 [95% CI 0.58–0.75] indicating substantial agreement. The users scored lower in confidence when interpreting test results that were weak positive cases (due to the relatively low signal intensity in the test-line) compared to strong positive cases. The end-users also found that the kit was easier to use than they expected (p < 0.001) and 231 of 264 (87.5%) reported that the test kit would meet their requirements if they needed an antibody testing kit. The overall findings provide an insight into the opportunities for improving the design of self-administered SARS-CoV-2 antibody testing kits for the general public and to inform protocols for future UX studies of LFIA rapid test kits.

## Introduction

Timely and accurate diagnostic testing plays an important role in preventing and controlling the spread of COVID-19. Several diagnostic techniques for SARS-CoV-2 have been recommended by the World Health Organisation (WHO)^1^: (1) detection of viral RNA via nucleic acid amplification tests (NAAT), such as real-time reverse transcription polymerase chain reaction (PCR); (2) detection of viral antigens through immunodiagnostic techniques, such as rapid diagnostic tests via lateral flow assays (LFIAs), and (3) detection of host antibodies through serological techniques, such enzyme linked immunosorbent assays (ELISAs). According to WHO^1^, NAAT is recommended as the reference standard since it is the most sensitive and specific; alternatively, rapid tests by LFIAs offer an opportunity to scale up testing capacity.

Given the importance of monitoring the presymptomatic or paucisymptomatic transmission of COVID-19^2,3^, large-scale community-based rapid testing^4,5^ becomes very important, which, however, is difficult to achieve by PCR testing because it can take at least one day^6^ or longer from requesting a test to receiving a result. Governments have invested enormous resources to scale up testing capacity and many countries have adopted the rapid diagnostic tests via LFIAs^5–9^. Although many studies have reported the performance of PCR^10–13^ and LFIA as a diagnostic tool for COVID-19^14–16^, there is a lack of user experience (UX) studies that investigate how the LFIA test kits are used by the general public for mass testing in different environmental settings, which is the gap in the literature that this study aimed to fill.

As well-established, low-cost, rapid and highly efficacious PoC devices, LFIAs have been developed for home pregnancy tests^17,18^, HIV^19,20^, Influenza A (H1N1)^21^, and more recently for COVID-19 antibody testing^22–26^. The UX studies for LFIA are commonly focused on evaluation for the accuracy in interpreting the test results and gathering the user response via questionnaire^17,19,23^. For example, a study^19^ for HIV self-testing based on 150 lay users conducting unsupervised self-testing aimed to assess whether the participants can correctly conduct all steps of the test. Results show that errors were found in the sample collection and transfer, as well as difficulties in interpreting the results. Another study^17^ investigated the usability and performance of seven visual home pregnancy tests that were available in Europe where each device claimed different sensitivity and accuracy scores. The study included 250 volunteers from the UK, who performed the test at home and at a study site. The usability study was evaluated by user scores based on 7-point Likert rating scales. Note these two UX studies were not for COVID-19 and they also did not cover the multidimensional aspects of UX analysis presented in this study.

There are limited studies involving user experience based on LFIAs for COVID-19^23,26^. A study for usability and acceptability^23^ was conducted for self-administered COVID-19 antibody testing in a home environment, which recruited 10,600 and 3800 participants in England for using two types of LFIAs. The presented usability analysis was summarised by descriptive statistics based on data from questionnaires, which identified the difficulties in the use of the lancet, and a need for clearer instructions for using the kit and interpreting the results. Agreement between the participant and a clinician’s interpretation of the results of the testing kits was assessed using Kappa scores and resulted in 0.72 and 0.89 scores for the two LFIA tests respectively. Another UX study for self-administrated SARS CoV-2 antibody testing kit was conducted in an in-car setting^26^ based on 1544 participants in Northern Ireland. The UX analysis based on 28 5-point Likert ratings from a post-test questionnaire suggested a good degree of UX and substantial agreement (Kappa score 0.75) in the interpretation of the test results by the participant and researcher. Analysis of the free-text responses in the survey suggests that the UX could be improved for blood-sample collection by modifying the method of sample transfer to the test device, and for interpretation of the results by giving clearer instructions.

The purpose of this UX study was to investigate the general public’s interaction with the LFIA testing kit at home, to identify the areas of difficulty encountered during testing, and to reveal valuable information and design opportunities for future improvement. The contribution of this study to literature includes three aspects: (1) Unlike other LFIA usability studies for COVID-19^23,26^ that were focused on the usability/acceptability and assessment of accuracy in interpreting test results, this current study carried out a more in-depth analysis, which covered the multidimensional aspects of UX including the ease of use, effectiveness, efficiency, accuracy (in interpreting the test results) and satisfaction. (2) To investigate how the participants perform in reading different test results, five types of LFIA test results were simulated and printed in the cards for participants to read. Using the simulated test results provides the controlled variation of test results, therefore, enabled us to gain a better understanding of how participants performed when reading different test results. (3) To assess various aspects of mental workload involved for home-based LFIA testing, National Aeronautics and Space Administration (NASA) task load index (TLX)^27^ was applied to estimate the user’s perceived cognitive demand of the task undertaken by the user. Workload assessment is valuable for the overall design of the test system as it may help to make the equipment and test procedure more user-friendly and potentially reduce error and improve effectiveness and customer acceptance.

The overall findings of this UX study will help the design of LFIA rapid testing for SARS-CoV-2 antibody and inform protocols for future studies, which may not only improve the LFIA testing performance by the general public but also have implications for coronavirus related public health planning.

## Materials and methods

### Test kit

The test kit used in this study was the AbC-19 Rapid Test developed by Abingdon Health, which has been approved for professional use in the UK and EU^28^ and has been used in another UX study for self-administrated LFIA for COVID-19 antibody testing in cars^26^. The AbC-19 Rapid Test is a single-use test for the detection of SARS-CoV-2 IgG antibodies in human capillary whole blood. Using a blood sample from a finger-stick puncture the test will identify the presence of IgG antibodies against the trimeric spike protein of SARS-CoV-2 virus (the virus responsible for the COVID-19 disease), signifying a recent or previous infection by the virus.

**Figure 1:**
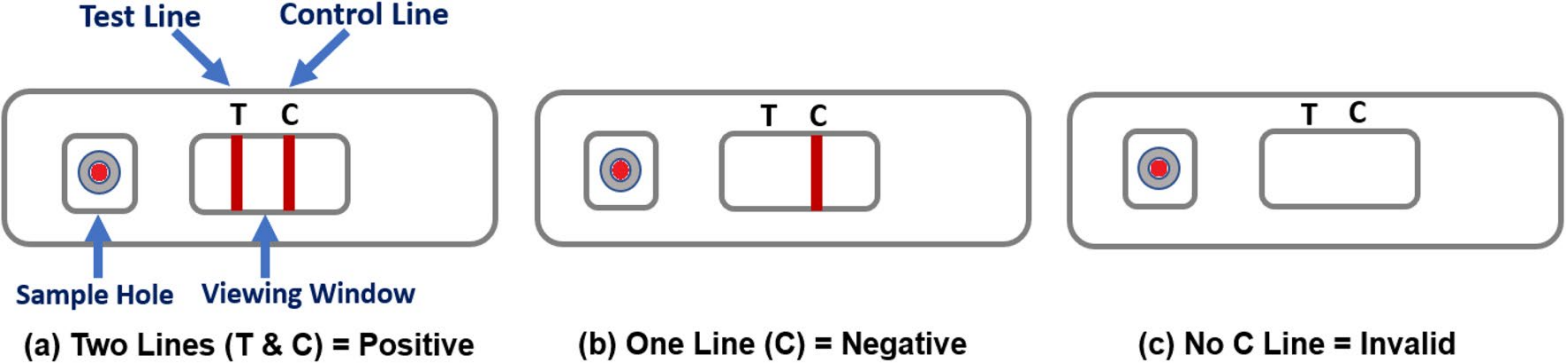
Illustration of the LFIA testing device: (**a**) structure of the testing device showing the sample hole, viewing window with the control line and test line as an example of positive result; (**b**) an example of negative result, and (**c**) example of invalid (without C-line).

The test kit materials include: one test, two single-use lancets, one blood collector, one test solution vial, a waste bag and instructions. The steps for performing the testing are outlined in the instructions (provided in Supplementary file): (a) hands were cleaned using warm water only; (b) the blood sample was taken from the ring or middle finger of the non-dominant hand by using the lancet; (c) the blood was collected using the blood collector; (d) blood was added to the test sample hole, and the test solution was then applied to the sample hole and the test allowed to develop; (e) after 20 min, if the C-line appears (indicating the test was performed successfully), the test results were interpreted by looking at the viewing window. Figure 1 illustrates the LFIA testing device and examples of results: (a) structure of the device showing the sample hole, viewing window with the control line (C-line) and test line (T-Line) as an example of positive result; (b) example of negative result; (c) example of invalid (without C-line).

### Participants

The home-based study was conducted from October to December 2020 in Northern Ireland (NI). The recruitment strategy was targeted email recruitment from our existing Pandemic Database. We targeted those with families and then a range of age, gender and education groups, which resulted in an over-recruitment of 55. From this 10 were excluded due to incomplete registrations. From the remaining groups, participants were selected based on age, gender, and education using the NI census data^29^ to achieve as balanced an overall cohort as representative of the NI population as possible. We had over-recruited middle-aged (31–50 years) females with a degree or higher and selected every 2nd entry—removing 45 participants.

Participants were invited to complete an online consent form and questionnaire via REDCap (www.project-redcap.org), which collected data such as gender, age, education, COVID-like symptoms, etc. The participants received Amazon vouchers after they completed the study. All participants were members of the public in NI and included children above the age of 7 years old and adults. Informed consent was obtained prior to commencing the study. Consent could only be given by individuals who were capable of independently understanding the information provided. Consent for children (< 18 years) was provided by their parent/guardian alongside child assent. Older participants and participants under 18 years old could be assisted by a family member or parent/guardian to complete the test and questionnaire.

### Study design

Difficulties in interpreting results appear to be one of the most common issues reported in UX studies for LFIA testing^19,20,22,23,26^. Our recent study for LFIA self-testing of SARS-CoV-2 IgG antibodies in cars^26^ suggested that the users found it difficult to interpret the results with the faint T-lines and hence resulted in false negative readings. One possible reason for the faint T-lines might be due to the dynamic changes in IgG levels among COVID-19 patients as some studies have suggested the rapid decay of anti-SARS-CoV-2 IgG in early infection^30–32^ and in the recovery stage^33^. Although most LFIA testing are qualitative and there is still a lack of quantitative studies for COVID-19 antibodies, a recent study^34^ has explored the quantitative analysis of LFIAs for early risk assessment of cardiovascular disease, in which the data show that the intensity of T-line changes according to eight different c-reactive protein (CRP) concentration levels.

**Figure 2:**
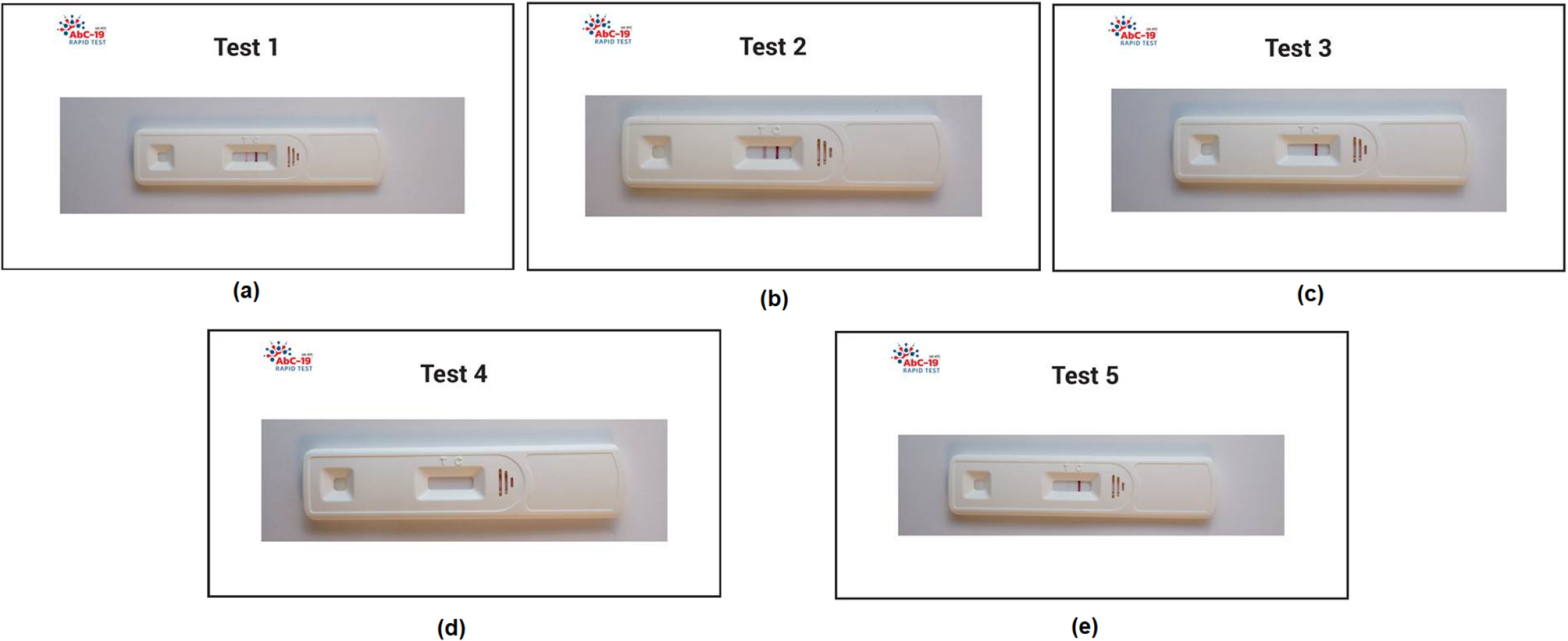
Examples of five printed test results that were designed for the study: (**a**) T1: positive; (**b**) T2: strong positive; (**c**) T3: negative; (**d**) T4: invalid, and (**e**) T5: weak positive.

To investigate how the participants performed in reading the test results with different T-line intensities, five simulated LFIA test results were printed in the cards, as shown in Fig. 2: (a) Test-1 (T1): positive; (b) Test-2 (T2): strong positive; (c) Test-3 (T3): negative; (d) Test-4 (T4): invalid and (e) Test-5 (T5): weak positive. The design of these five LFIA was based on the findings from our recent study for LFIA self-testing conducted in cars^26^, in which the false negative results comprised mostly of faint test lines (like T5). The faint T-line may be reflective of the level of antibodies present in the blood of subjects who may have been infected in March 2020 during the first wave of COVID-19 within Northern Ireland whilst that study was conducted almost 6 months later. Using the simulated test results helps to provide the controlled variation of T-line intensity, which enabled us to gain a better understanding of how participants performed when reading different test results.

**Figure 3:**
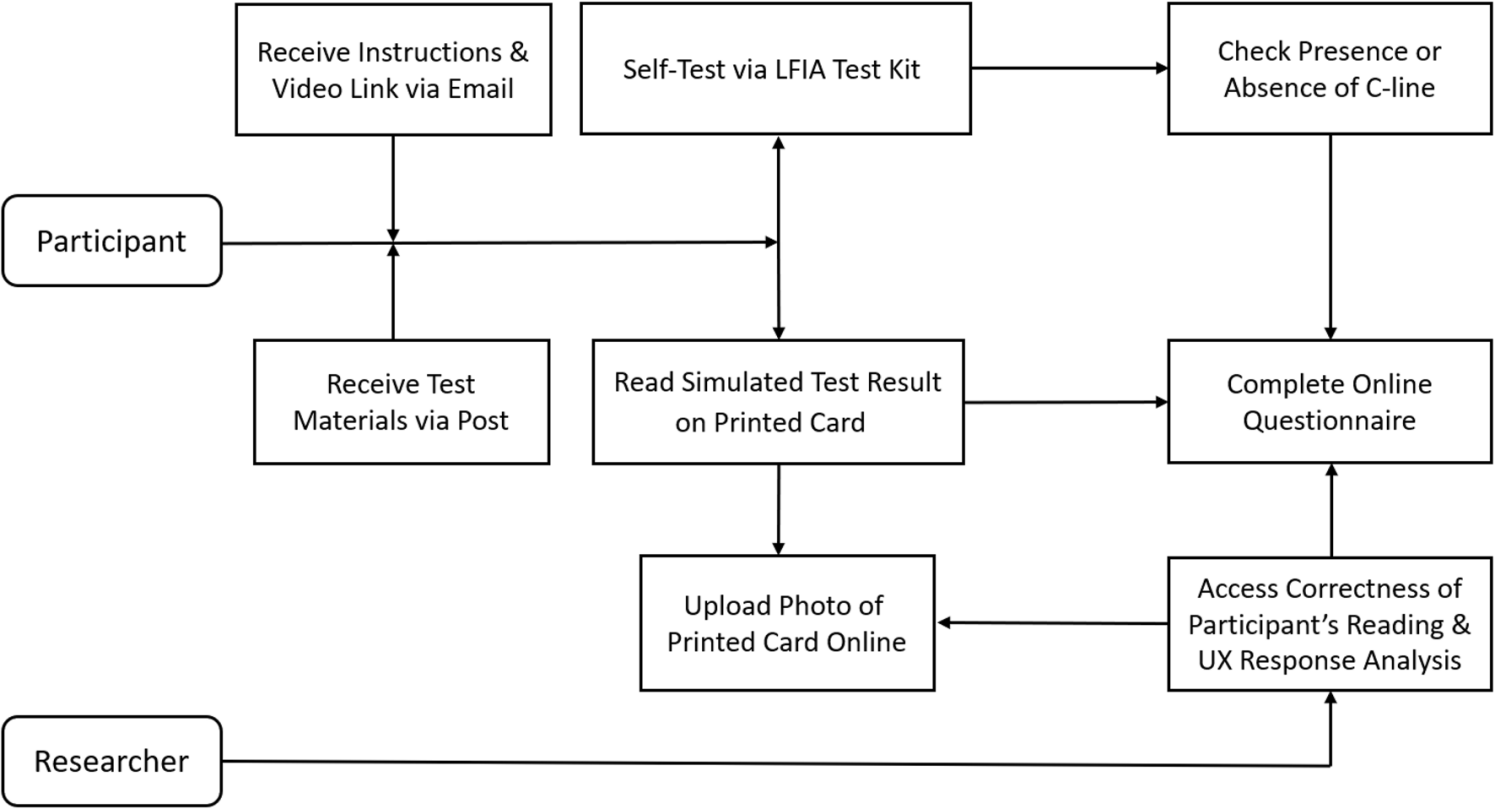
The diagram for the study design.

The diagram for the study design is presented in Fig. 3. Participants received the test kits via post and were given prior access to written instructions and a video on YouTube^35^ before using the test. Participants were asked to follow the instructions provided and to complete three tasks: (1) apply blood sample to the testing kit, wait for the C-line to develop and answer whether they have observed the C-line in the test, and upload a photo of their finished test. Note the purpose of this task was not to test for the actual existence of the COVID-19 antibodies but to assess the participants’ ability to complete the test successfully via observing the presence of C-line and identifying the reasons for failure and areas of difficulties. Therefore, the participants needed to follow all steps in instruction to apply the test to themselves to develop the C-line. (2) interpret the test result printed on a card, which was randomly selected for them from five simulated test results (as in Fig. 2) and choose the reading that most closely matched their interpretation from the four options provided: positive, negative, failed/invalid and unsure. They were also asked to upload the photo of their printed card, which was used by the researchers to assess the correctness of the participants’ readings via comparing to the ground truth; (3) complete the post-test questionnaire online. Note, assessment of participants’ reading for actual LFIA testing results against the clinicians’ or researchers’ results have been done in two related studies at home^23^ and in cars^26^, which was not the purpose of this study. Therefore, the participants’ reading against ground truth (in printed cards) was used in this study.

### UX analysis

The data regarding the UX of the testing kit were collected from the post-test questionnaire (provided in Supplementary Information). The questionnaire was based on modification and extension of the recent UX study for LFIA self-testing in cars^26^. To simplify the task for the end-users, the 5-point Likert rating questions used in the previous study^26^ were replaced by polar questions. Furthermore, two new question sections were added for assessment of TLX^27^ and satisfaction.

The questionnaire comprised of 13 sections and each section entailed 3 to 6 questions, which included 30 polar questions and 11 Likert rating questions in total. There were 27 of 30 polar questions measured the UX of a particular aspect of the testing kit, which include: (Q1) outer packaging; (Q2) collection of finger prick blood sample; (Q3) application of sample to test; (Q4) application of test solution to LFIA device; (Q5) development of a control line and interpretation of results; (Q6) instructions for use; (Q7) risks and warnings. The remaining 3 polar questions were focused on the completion of test (Q8a), whether participants received help (Q9c) and interpretation of the results (Q10c). There were 11 7-point Likert rating scales in the two sections (Q11 and Q12). Q11 was designed for obtaining the task load index (TLX)^27^ and Q12 was for assessing the comfort and acceptability of the end-users for the test kit. The presentation of the UX analysis was organised in five areas: (1) ease of use; (2) effectiveness; (3) efficiency; (4) accuracy and (5) satisfaction, which are explained in detail next.

1. *Ease of use* Ease of use was evaluated based on the UX and usability scores, a percentage of ratings from the polar questions in Q1–Q7, by counting “Yes” from all answers then normalised to 100. The summative scores (sum of scores normalised as a percentage) for all participants in each section were calculated and analysed. Although there are diverse definitions of UX, most agree that UX is more than just a sum of a product’s usefulness and usability^36–38^. Both UX and usability were analysed using the same approaches in our early study^26^, in which UX was considered a higher level construct (where usability is a sub-component). All polar questions in Q1–Q7 (except Q2e and Q6b for efficiency) were used for calculating UX scores. Questions not describing the usability constructs, Q1a, Q2d, Q4d, Q7a, Q7b, Q7c, Q7d and Q7e were removed for the usability analysis.
2. *Effectiveness* ISO9241 defines effectiveness as “the accuracy and completeness with which specified users can achieve specified goals in particular environments”, hence effectiveness was assessed by the test completion rate. A presence of C-line within the test window indicates a successful completion of test. The number of users who completed the test (via achieving a C-line) was examined and the reasons for failure and areas of difficulty to complete the test were analysed.
3. *Efficiency* ISO9241 defines efficiency as “the resources expended in relation to the accuracy and completeness of goals achieved”. Here efficiency was assessed in the following aspects: physical resource, human support and mental workload. For physical resource, feedback was gathered on the users’ responses to whether the second lancet was used (Q2e), how usable the instructions were (Q6b) and the frequency of consulting instructions (Q6f). For human support, the percentage of those who requested help from others was obtained and analysed (Q9c). NASA’s TLX was applied to assess the mental workload. Independent studies have found TLX to be a valid measure of subjective workload^27,39,40^ and has become the gold standard for measuring subjective workload across a wide range of applications from healthcare^41–43^ to technology domains^44^. TLX is a six-item scale and each item represents a different aspect of workload: Mental Demand (MD), Physical Demand (PD), Temporal Demand (TD), Effort (EF), Performance (PE) and Frustration (FR). Research has shown that the raw TLX (without weighting the contribution of each factor in a predefined manner) has a high correlation with the weighted one^45^, but is more time efficient and simpler to apply^46,47^, therefore no weighting was applied for TLX in this study. To aid in interpretation and simplify use, the items are combined into a single summed unweighted score representing the latent construct of overall workload experienced by the individual during a specific time, event or situation^44,46^. Like previous studies^46,48^, we simplified the original 21-point TLX Likert scale questions to 7-point. Like many studies based on raw TLX^49^, all ratings for six subscales were normalised to 100 and the final TLX score was the mean of six subscale ratings. We further investigated whether there are differences in mental workload for people of different ages and education levels. Like another study^26^, we categorised the participants into four age groups (as shown in Table 1, 7–17, 18–30, 31–60 and age above 60) and four educational attainments (Master/PhD, Honours Degree, A-level/NVQ (National Vocational Qualifications) and Primary/Secondary/Other education). For every group, the TLX scores were calculated and the pair-wise Wilcoxon tests were performed between the groups.
4. *Accuracy* For clarity, the focus of this study was not to address or report on the diagnostic accuracy of the LFIA test to detect antibodies against SARS-CoV-2 virus^50^, instead, the focus was to assess the user experience of the test kit. The simulated LFIA test results were used, which provided the controlled variation of T-line intensity and enabled us to gain a better understanding of how participants performed when reading the test results. The correctness of participants’ reading for the printed test results were assessed by researchers by comparing to the ground truth. The accuracy was measured based on the agreement rate and Kappa score. Further investigation was carried out on which types of test results the members of the public most often misinterpreted.
5. *Satisfaction* ISO 9241 defines satisfaction as “the comfort and acceptability of the work system to its users and other people affected by its use”. The feedback on satisfaction was gathered in Section Q12. Similar to a recent UX study^23^ based on self-testing for COVID-19 at home, in which the acceptability was assessed by the end users’ willingness to perform finger-prick antibody tests, the question used in this study was to assess whether the users think the test kit meets their requirements (Q12a). Furthermore, to assess the comfort, questions asked the end users’ perceived ease of using the test kit before and after testing (Q12b and Q12c) and the confidence in both completing the LFIA test and reading the test results (Q12d and Q12e).

### Statistical analysis

The Chi-square test was applied to assess the differences between two proportions. Wilcoxon rank-sum tests were applied for data that were not normally distributed as indicated by the Kolmogorov–Smirnov-test. Bonferroni correction^51^ was applied to adjust the significance level $\alpha$ during multiple hypothesis testing. The effect size for Chi-square test was measured by $\phi$ coefficient proposed by K. Pearson^52^, The effect size $\eta ^2$ for Wilcoxon rank-sum tests was calculated using the z-scores of rank-sum tests as proposed by Cohen^53^. The effect size for t-test was calculated based on Cohen’s *d*^54^.

The agreement rate was assessed by the percentage of cases where the results interpreted by the participants’ agreed with the printed test results (the ground truth). Kappa statistics^55–57^ was also applied to evaluate the agreement, which was the metric of choice in COVID-19 antibody self-testing study^23,26^ and HIV rapid diagnostic tests^58–60^. The range of Kappa scores can be interpreted as follows^61^ : < 0 = poor agreement, 0.00–0.20 = slight agreement, 0.21–0.40 = fair agreement, 0.41–0.60 = moderate agreement, 0.61–0.80 = substantial agreement, and > 0.8 = almost perfect agreement.

All data analyses were performed using MATLAB2019b (MathWorks, USA) and Microsoft Excel for Microsoft 365 (MSO 32-bit).

### Ethical approval

This study was approved by Ulster University Research Ethics committee (Ref: REC/20/0043) in full adherence to the Declaration of Helsinki. All participants provided fully informed consent. Informed consent for children (<18 years) was obtained from parents/guardians alongside assent from the child.

## Results

### Characteristics of study participants

**Figure 4:**
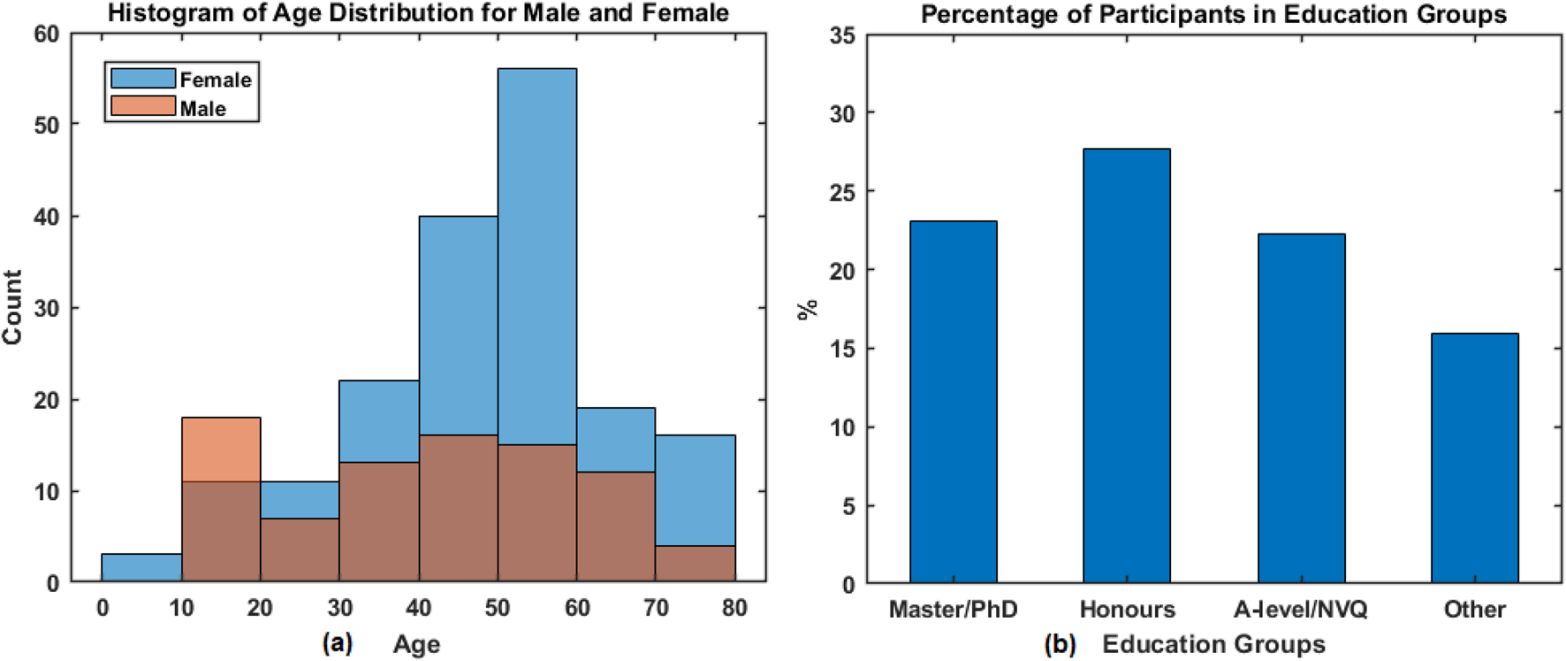
(**a**) The histogram of age distribution for female and male participants; (**b**) percentage of participants in four education groups.

There were 264 individuals participated in the study and the characteristics of the participants are provided in Table 1, which presents the proportion of participants in gender, age, education and ethnicity. The histograms of age distribution (for male and female) are presented as in Fig. 4a. It is noticed that female participants were slightly over-represented (as also shown in Table 1 that 178 of 264 (67%) participants are females). The percentages of participants in four educational groups are shown in Fig. 4b, which shows less participants in Primary/Secondary/Other education (shown as ‘Other’) when compared to the other groups. However, every effort has been made to achieve as balanced an overall cohort as possible.

**Table 1:** Characteristics of study participants. The values for age are presented in the range, mean ± standard deviation (SD).

Characteristics	Groups	Proportions n (%)
No. of participants	All	264 (100)
Gender	Male	85 (32.2)
	Female	178 (67.4)
	Prefer not to say	1 (0.4)
Age [7–79, 45.0 ± 17.4]	7–17 (12.9 ± 2.5)	30 (11.3)
	18–30 (24.7 ± 3.8)	26 (9.9)
	31–60 (47.4 ± 7.7)	160 (60.6)
	60 + (68.3 ± 5.3)	48 (18.2)
Education	Ph.D	13 (4.9)
	Master	48 (18.2)
	Honours degree	73 (27.7)
	A-Level /level3 NVQ/diploma	59 (22.3)
	Secondary School Education	32 (12.1)
	Some Secondary Education	1 (0.4)
	Other Education	9 (3.4)
	Missing	29 (11.0)
Ethnicity	White	262 (99.2)
	Other	2 (0.8)

### Ease of use

**Figure 5:**
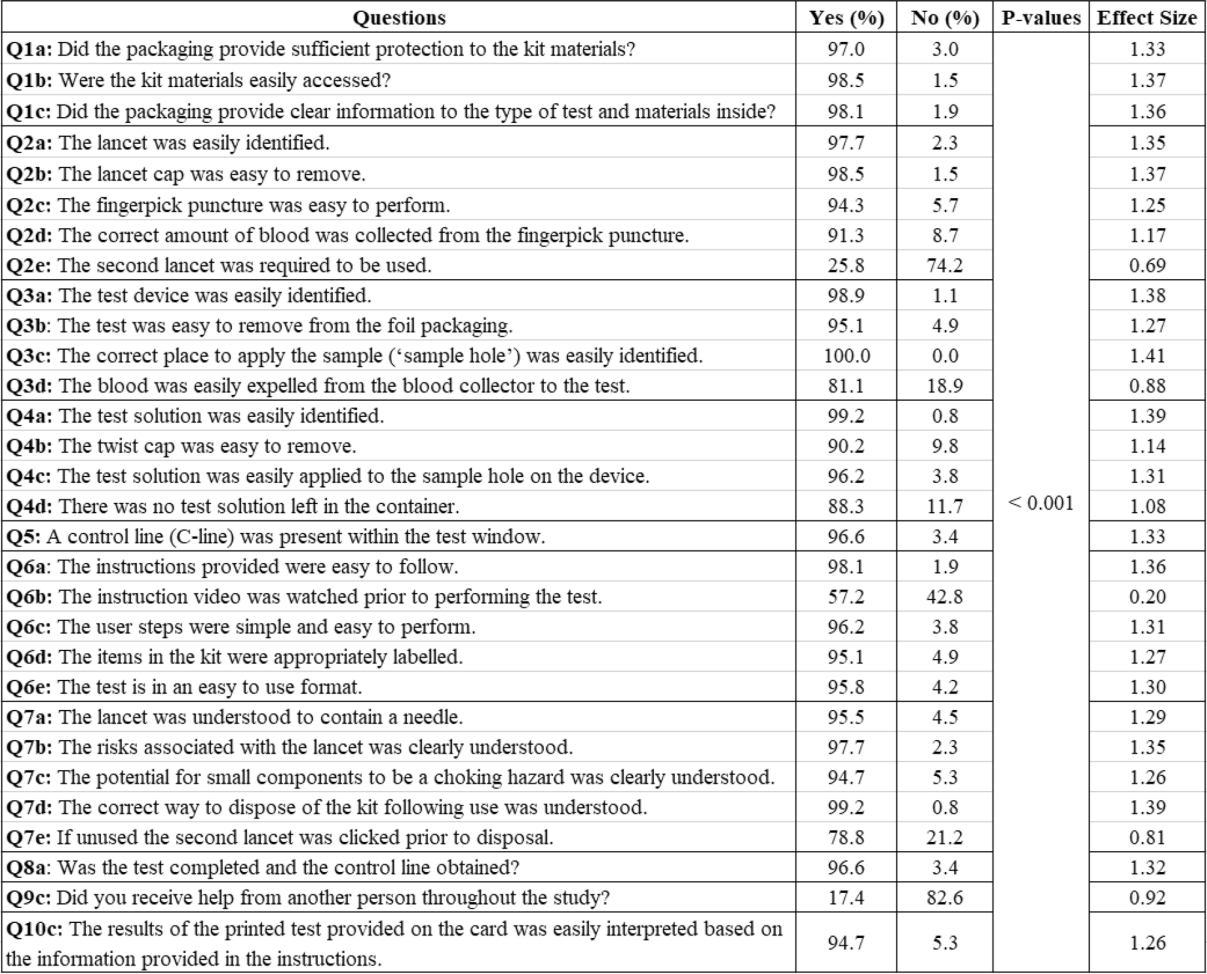
Summary of participants’ responses to polar questions survey together with p-values and effect size $\phi$.

There were 30 polar questions in ten sections in the questionnaire, the participants’ answers for each polar question were counted. The percentage of the counts are provided in Fig. 5. The p-values from Chi-square tests suggest statistical significance ($p < 0.001$) and the effect sizes suggest medium or large effect except for Q6b ($\phi =0.20$ indicating small effect).

**Figure 6:**
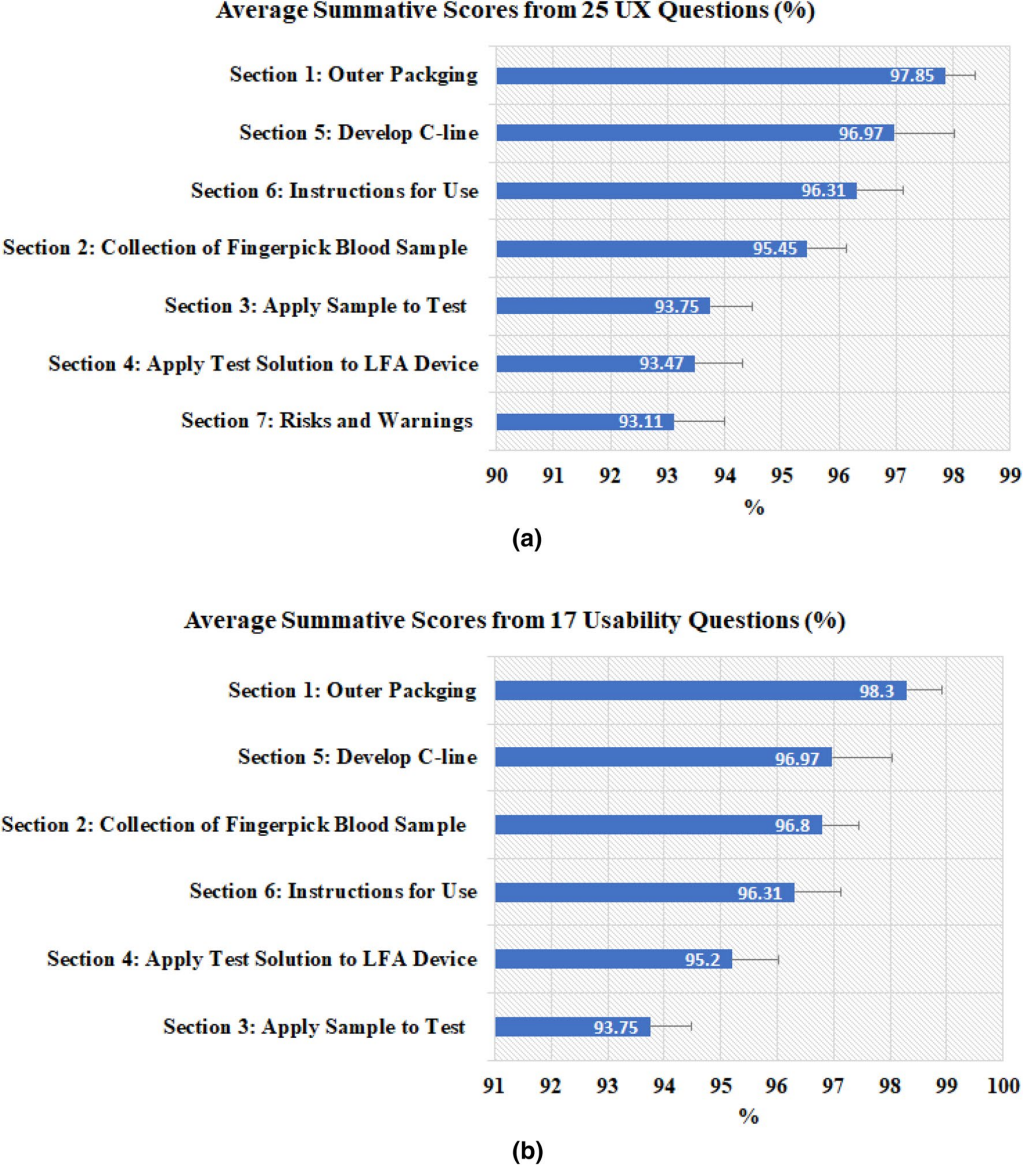
Average summative scores based on: (**a**) 25 UX questions; (**b**) 17 questions related to usability construct only. The error bars represent the standard errors (n = 264).

The sections Q1 to Q7 were devoted to specific aspects of the testing kit and used to assess the ease of use. The summative UX scores (sum of scores normalised as a percentage) for 264 participants from Q1 to Q7 were calculated (in which Q2e and Q6b were excluded and analysed for efficiency), and the mean and standard errors are presented in Fig. 6a. With the mean of each section higher than 93.11% and an overall average 95.27% (95% CI 92.71–97.83%), the public found the test kit relatively easy to use. Figure 6a also suggests the areas that could be improved, i.e., the application of test solution to LFIA device (Q4). A relatively low score was found in Q7e with 78.8% confirming that the unused second lancet was clicked prior to disposal, which suggests more effort will be needed to increase the users’ awareness of the risks of unused lancet.

Similar to the approach used in another study^26^, to assess the usability, we removed the questions Q1a, Q2d, Q4d, Q7a, Q7b, Q7c, Q7d and Q7e because they were not related to the usability construct. The summative usability scores were then calculated for the remaining 17 questions. The results of usability scores with mean and standard errors are given in Fig. 6b. The means for each section are higher than 93.7% with an overall average of 96.2% (95% CI 93.8–98.5%).

It can be seen in Fig. 6a that the scores for section Q2 (relating to collection of the blood sample) and section Q4 (relating to application of test solution to LFIA device) are lower than those in Fig. 6b. The reason for this was due to Q2d and Q4d being excluded from calculating usability scores. From Fig. 5, we can see that participants scored relatively low (91.3%) for Q2d (‘The correct amount of blood was collected from the finger prick puncture’) and 81.1% for Q3d (‘The blood was easily expelled from the blood collector to the test’), which suggests that these were the barriers to optimal user experience. Q4d asked if there was any test solution remaining in the container, which has a relatively low score of 88.3%, which suggests that some users might not realise that they should have used all of the test solution.

### Effectiveness

**Figure 7:**
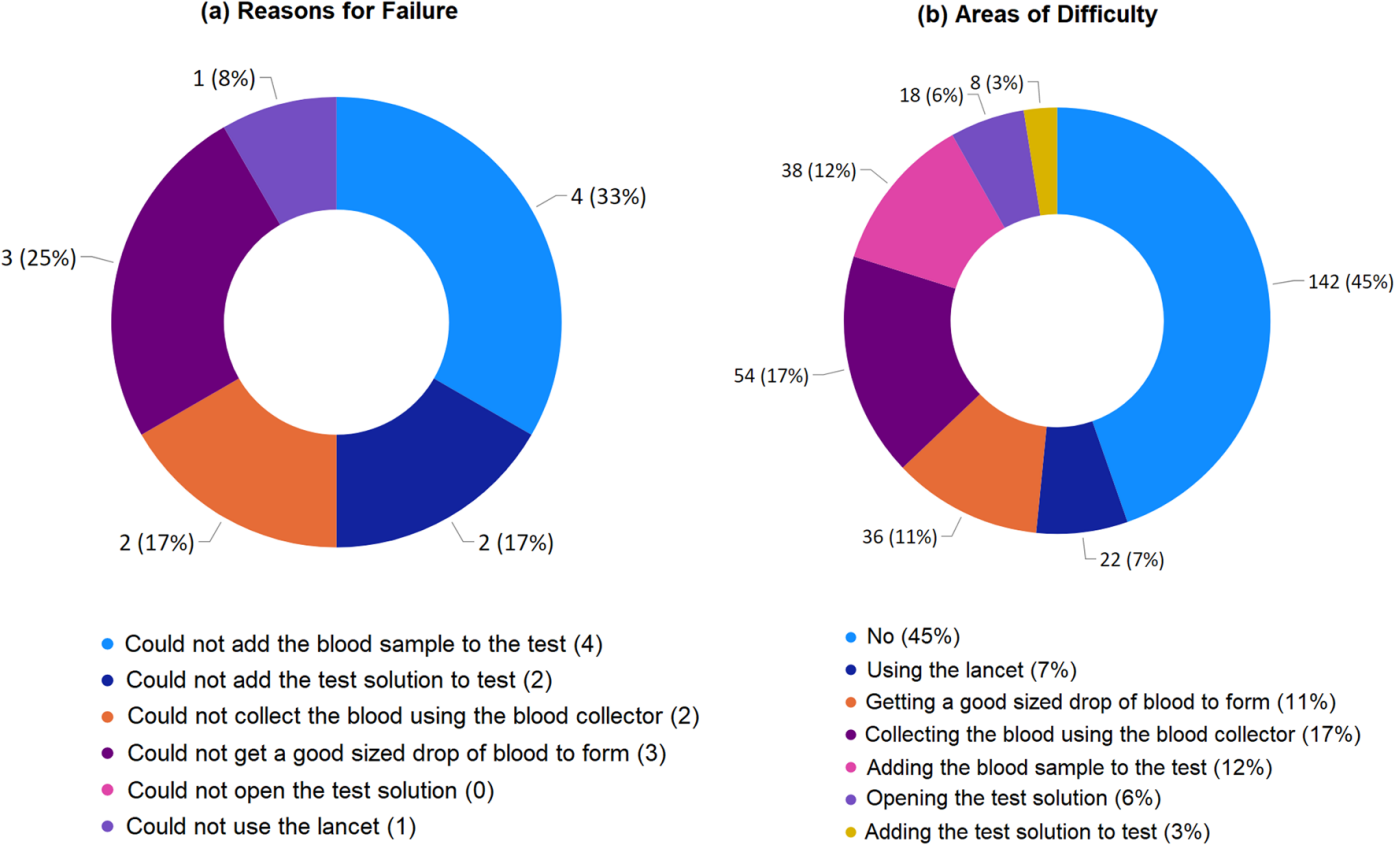
Summary for: (**a**) reasons for failure to complete the test based on 12 multiple choices; (**b**) areas of difficulty during the test based on 318 multiple choices.

Effectiveness was assessed by test completion rate (Q5), in which 255 of 264 (96.6%) participants successfully completed the test after obtaining the control line, suggesting a good degree of effectiveness of the test kit. There were 9 participants for whom the test failed, in which 1 of 9 (11.1%) came from the age 18-30 group, 6 of 9 (66.7%) from age 30–60 group and 2 of 9 (22.2%) were aged over 60. The reasons for failure (Q8b) with multiple choices are summarised in Fig. 7a. The most common reason for failure was failure to add the blood sample to the test followed by failure to get a sufficiently sized drop of blood to form.

All participants also identified other areas of difficulty in their experience that did not lead to a test failure. There was a total of 318 choices received from 264 participants and the proportion in total choices is shown in Fig. 7b. It can be seen that 142 of 318 (45%) responders had no difficulty in completing the test, however the most difficult part of the test, as reported by 54 of 318 (17%) was collecting the blood using the blood collector. This was followed by 38 of 318 (12%) who said that their difficulty was adding the blood sample to the test, which was also reported as the main reason for failure.

### Efficiency

1. *Physical resource* The response to Q2e shows that 74.2% participants completed test using only one lancet suggesting user efficiency without the need for the spare lancet (each test kit included two lancets). Regarding the instruction video, answers to Q6b indicate that 57.2% of users watched the instruction video prior to performing the test, yet according to answers for Q5, 96.6% of users completed the test successfully, which suggests that the procedure was relatively easy to understand and implement even if not all users watched the video. For 9 users who failed the test, 6 of them watched the video before test, which may suggest that watching the video or not may have no direct impact on the failure of the test.

Question Q6f asked how many times the participant consulted the instructions during the test. As seen in Table 2, the majority of participants, 214 of 264 (81.1%) consulted the instructions 1–3 times, 42 of 264 (15.9%) consulted instructions 4–6 times, 5 participants consulted the instructions 7–9 times and only 3 consulted instructions more than 10 times. Among the 3 people who consulted the instructions at least 10 times and 5 people who consulted instructions 7–9 times, none of them were over 60 years old. Among 214 who consulted instruction 1–3 times, no significant difference was found between four age groups (as shown in Supplementary Table S1).

We cross examined the answers from the participants who watched the instruction video vs those who did not. From Table 3, it shows that among the 214 who consulted the instruction 1–3 times, 87 of 214 (40.7%) did not watch the video, which is the lowest proportion in the four categories; 5 of 5 (100%) and 2 of 3 (66.7%) that didn’t watch the video consulted the instructions 7–9 times and more than 10 times, respectively. It appears that the higher the proportion of users who didn’t watch the video, the more times they consulted the instructions, which indicates the effectiveness of the video in informing participants.

**Table 2:** Number of times the participants consulted the instructions in four age groups.

Number of times users consulted the instructions	Age 7–17	Age 18–30	Age 31–60	Age 60+	Total
1–3	23	22	131	38	214 (81.1%)
4–6	5	2	25	10	42 (15.9%)
7–9	1	1	3	0	5 (1.9%)
10 +	1	1	1	0	3 (1.1%)
Total	30	26	160	48	264 (100%)

**Table 3:** Number of times the participants consulted the instructions for those watched the instruction video and those did not.

Number of times users consulted the instructions	Watched video	Didn’t watch video	Total
1–3	127	87	214
4–6	23	19	42
7–9	0	5	5
10 +	1	2	3

(2) *Human support (help received)* The question Q9c asked whether the participants received help from another person during the study. The answers suggest that 218 of 264 (82.6%) completed the test independently without another person’s help and of 46 of 264 (17.4%) requested help from others. We examined the participants who requested help in each age group and results are given in Table 4. The table shows that 21 of 30 (70%) in the age group 7–17 requested help from others, which is significantly higher than the rest three age groups (p-values < 0.001 and effect size $\phi > 0.46$ as presented in Supplementary Table S2). There are 11 of 48 (23%) from the age group 60+ also requested help, which is significantly higher than those in age group 31–60 (p = 0.003, effect size $\phi =0.21$). The results suggest that additional support is needed for these two age groups (< 18 and 60+) during the self-administrated test.

**Table 4:** Number of participants in four age groups that received help from others during the test.

Received help	Age 7–17	Age 18–30	Age 31–60	Age 60 +	Total
Yes	21 (70%)	2 (8%)	12 (8%)	11 (23%)	46 (17%)
No	9 (30%)	24 (92%)	148 (92%)	37 (77%)	218 (83%)
Total	30	26	160	48	264

**Figure 8:**
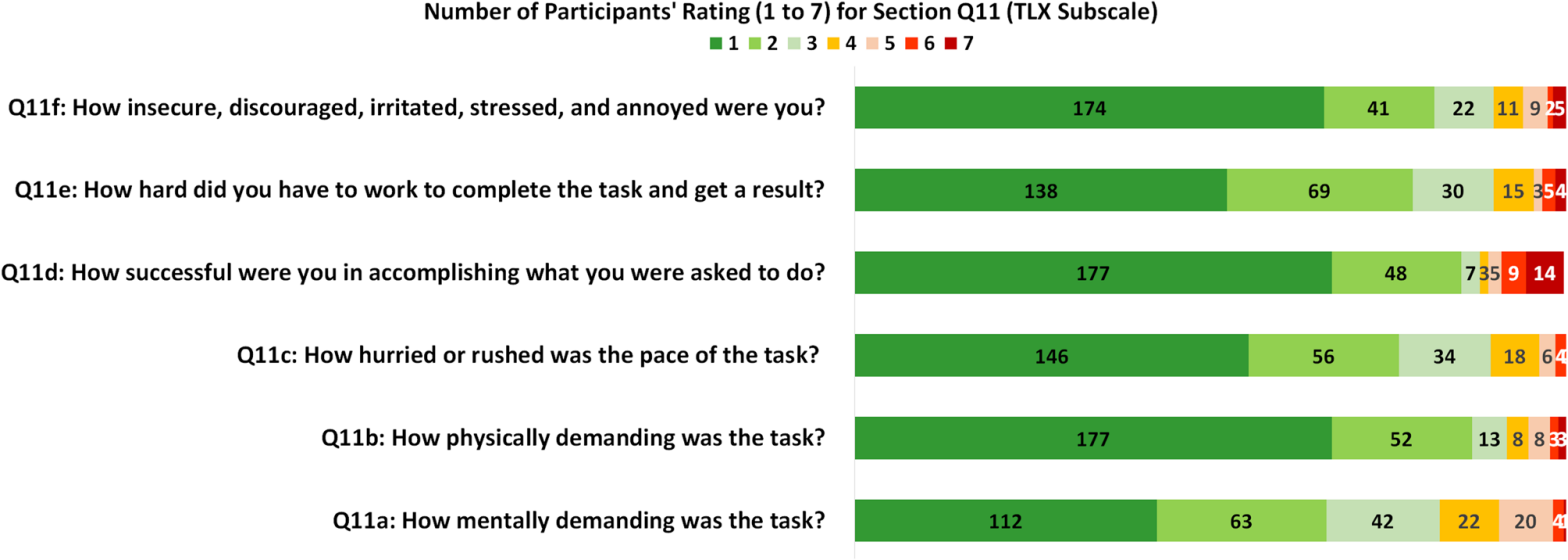
Summary of number of participants’ rating in six TLX subscales.

**Figure 9:**
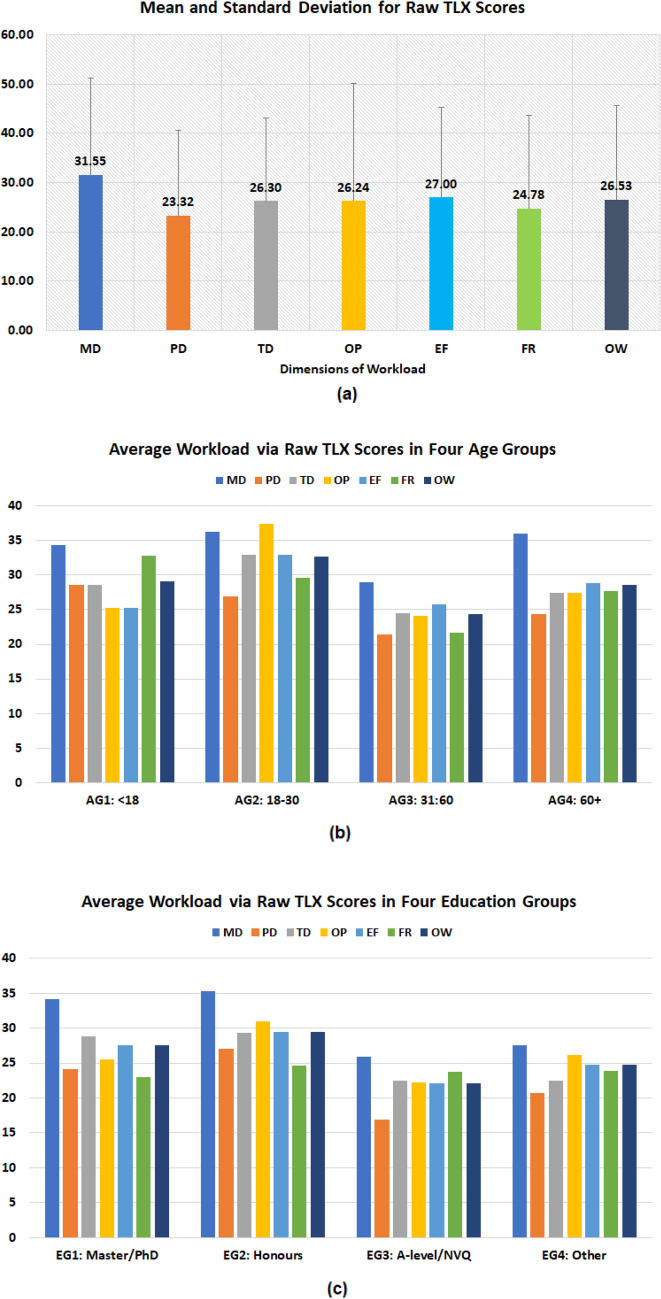
Results of TLX and six subscales: (**a**) mean and standard deviation based on all participants (n = 264); (**b**) mean based on participants from four age groups (AGs); (**c**) mean based on participants from four education groups (EGs).

(3) *Mental workload* The counts for six TLX subscales are presented in the Fig. 8, in which each item has a scale rating from 1 to 7. The results of mean and SD of TXL scores and six workload subscales (n = 264, normalised to 100) are provided in Fig. 9a, in which the mental demand (MD) has the highest score (31.5 ± 19.7), followed by Effort (EF) (27.0 ± 18.3), temporal demand (TD) (26.3 ± 16.8), own performance (OP) (26.2 ± 23.8), frustration (FR) (24.8 ± 18.9), and physical demand (PD) has the lowest score (23.3 ± 17.2). The overall workload TLX score is 26.5 ± 19.1.

A further investigation was carried out to study the mental workload for four age groups (AGs) and four education groups (EGs). Fig. 9b presents a bar chart for the average of six TLX subscales from the participants in four age groups. The results show that among all factors, mental demand (MD) appears to score the highest in three of four groups, which suggests that the participants considered the task more mentally demanding when compared to other factors. The age group AG4 (60+) gave the highest score for mental demand and AG3 (31–60) has the lowest MD score. The three groups (AG1, AG2 and AG4) scored high for physical demand (PD) and frustration (FR). But AG4 (60+) and AG1 (< 18) did not score the highest for temporal demand (TD), perhaps due to both groups received helps from others (as shown in the results in Table 4). For the overall workload TLX, the AG3 (31–60) has the lowest score, which suggests that the AG3 considered the test having less mental workload when compared to the other AGs. Fig. 9c presents the bar chart for mean of six TLX subscales for the participants in each of the four EGs. Similar to that in the age groups, mental demand (MD) appears to score the highest among all four groups. For the overall TLX scores, the EG2 (Honours) appears to score higher than the rest and EG3 (A-level) has the lowest TLX score.

One-sample Kolmogorov–Smirnov tests were performed for each group and the results suggest the scores do not have a standard normal distribution. The paired Wilcoxon rank sum tests were applied to access the medium differences between the groups. The significance level $\alpha$ was set as 0.008 via Bonferroni corrections. The results of p-values and effect size $\eta ^2$ based on the age groups are presented in Table 5 and no significant differences were found in the paired comparison. The results for education groups are given in Table 6, EG1 (Master/PhD) and EG2 (Honours) appear to score significantly higher than EG3 (A-levels) but both have small effect sizes. No statistical significance was found for other paired tests. Overall, the average TLX for each group is lower than 30 indicating a relatively low level of mental workload compared to the reported reference values for TLX^49^ in the domain of manual labour and when using handheld devices .

**Table 5:** p-values and effect size $\eta ^2$ (in bracket) based on paired Wilcoxon tests for TLX scores in four age groups.

Age groups	AG1 7–17	AG2 18–30	AG3 31–60	AG4 60+
AG1 7–17	1 (0)	–	–	–
AG2 18–30	0.372 (0.119)	1 (0)	–	–
AG3 31–60	0.320 (− 0.072)	0.026 (− 0.164)	1 (0)	–
AG4 60 +	0.824 (0.025)	0.382 (− 0.102)	0.092 (0.117)	1 (0)

**Table 6:** p-values and effect size $\eta ^2$ (in bracket) based on paired Wilcoxon tests for TLX scores four education groups.

Education groups	EG1: Master/PhD	EG2: Honours	EG3: A-Levels	EG4: Other
EG1: Master/PhD	1 (0)	–	–	–
EG2: Honours	0.773 (0.025)	1 (0)	–	–
EG3: A-Levels	0.002 (− 0.281)	0.002 (− 0.265)	1 (0)	–
EG4: Other	0.088 (− 0.168)	0.069 (− 0.170)	0.470 (0.072)	1 (0)

### Accuracy: agreement between participant-interpreted and printed results

The results interpreted by the participants were compared to the ground truth from the printed cards. The summary of the results is presented in Table 7. The rows of the table are the participants’ results (via answers to Q10b) in four categories: positive, negative, invalid and unsure. The columns show their printed cards that can be one of the five categories: positive (T1), strong positive (T2), weak positive (T5), negative (T3) and invalid (T4). The column of ‘Total Positive’ is the sum of all positives (T1, T2 and T5). The agreement rate between participants’ results and the ground truth (based on the counts for total positive, negative and invalid) is 80.63% [95% CI 75.21–86.05%], and the Kappa score is 0.67 [95% CI 0.58–0.75] which suggests a substantial agreement between the results interpreted by the participant and the ground truth.

**Table 7:** Results read by the participants and the ground truth provided in the printed cards. The values in the brackets show the percentage of the results related to total number of results (n = 264).

		Printed cards’ results n (%)						
		Positive (T1)	Strong positive (T2)	Weak positive (T5)	Total positive	Negative (T3)	Invalid (T4)	Total
Participant results: n (%)	Positive	43 (16.3)	29 (11.0)	48 (18.2)	120 (45.5)	2 (0.8)	1 (0.4)	123 (46.6)
	Negative	14 (5.3)	8 (3.0)	13 (4.9)	35 (13.3)	59 (22.3)	7 (2.7)	101 (38.3)
	Invalid	0 (0.0)	0 (0.0)	2 (0.8)	2 (0.8)	2 (0.8)	25 (9.5)	29 (11.0)
	Unsure	1 (0.4)	0 (0.0)	1 (0.4)	2 (0.8)	1 (0.4)	8 (3.0)	11 (4.2)
	Total	58 (22.0)	37 (14.0)	64 (24.2)	159 (60.2)	64 (24.2)	41 (15.5)	264 (100)

As seen in Table 7, there were 35 False Negative (FN) cases which the participants interpreted the results as negative, but the printed cards were positive. There are 13 of 35 (37.14%) FN cases were ‘weak positives’ (T5) and 14 of 35 (40%) FN cases were positive (T1), but only 8 of 35 (22.85%) cases were ‘strong positive’ (T2) cases and 7 of 35 (20%) were invalid (T4) cases. This suggests that results from the categories of positive (T1) and weak positive (T5) were more difficult to interpret due to the relatively low signal intensity in the test-line when compared to the strong positive (T2) cases.

### Satisfaction

**Figure 10:**
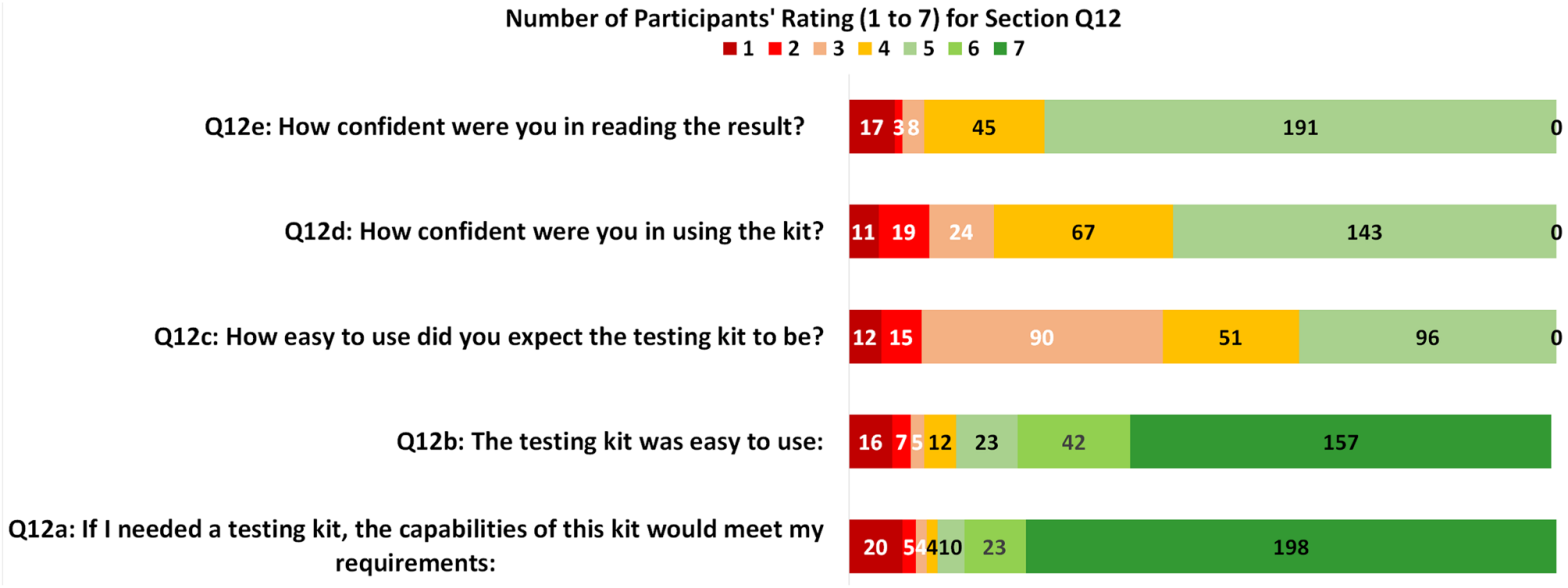
The counts for the 7-point Likert rating scores for question section Q12 (which helps to assess the comfort and acceptability).

The answers to Q12 helped to assess the comfort and acceptability (the satisfaction) the users perceived for the test kit and the results for the rating counts are summarised in Fig. 10. The answers for Q12a show that 231 of 264 (87.5%) scored over 5, which suggest that majority of users considered the capability of the test kit meet their requirements.

**Figure 11:**
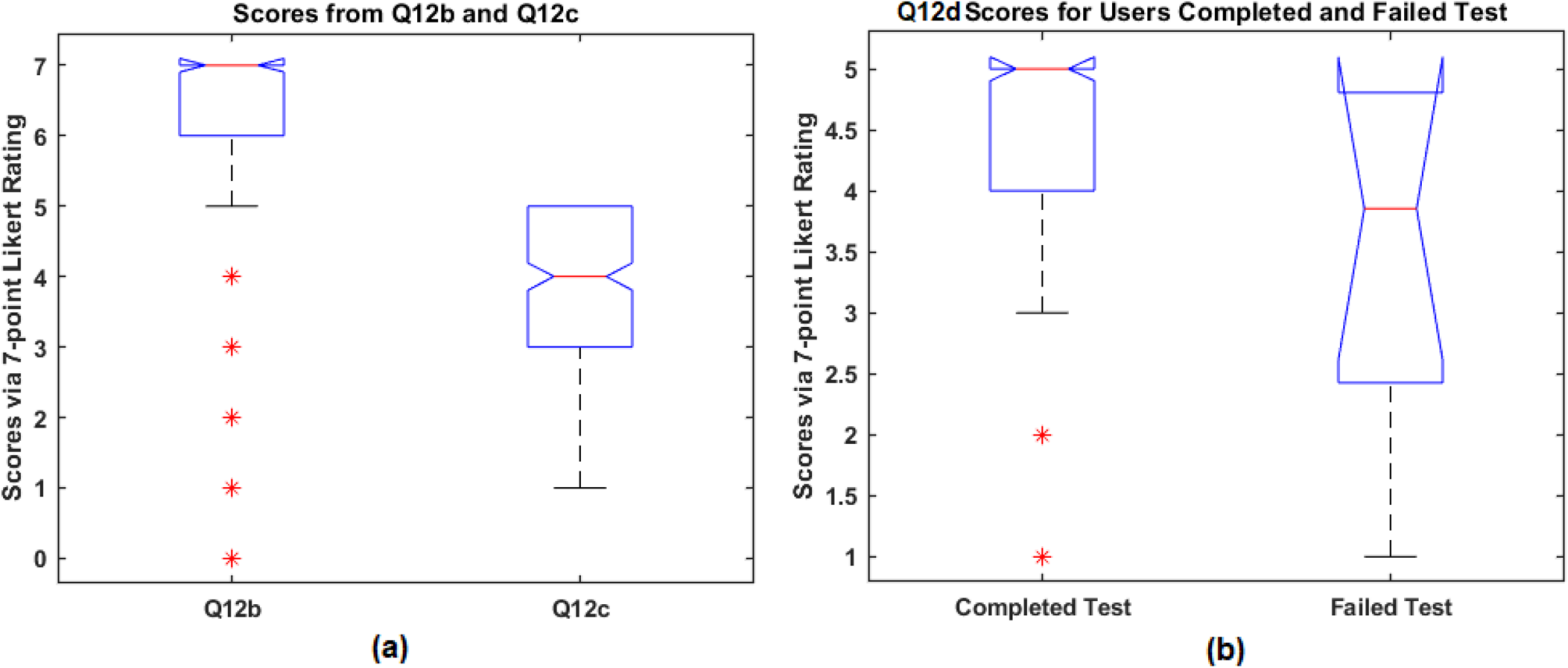
The boxplot for: (**a**) scores from Q12b and Q12c to assess the user-perceived ease of using the kit after and before the test; (**b**) scores for Q12d (to assess the confidence in using the kit) from the users who completed and failed the test.

The scores from Q12b and Q12c were the responses on the user-perceived ease of using the kit after and before the test, respectively. A boxplot based on the scores from Q12b and Q12c are given in Fig. 11a. The higher median of Q12b than Q12c suggest that the participants found the kit was easier to use than they expected (p < 0.001, effect size $d=1.41$).

In terms of the confidence in using the kit (Q12d), as seen in Fig. 10, the highest score was 5 received from 143 of 264 (54.2%), 67 of 264 (25.4%) scored 4, which suggest the relatively low confidence the users felt in using the kit. However, the answers to Q5 show that 255 of 264 (96.6%) users successfully completed the test, which suggests that even the participants who felt a lack of confidence still managed to complete the test. A boxplot for Q12d scores from those who completed and failed the test is given in Fig. 11b, which shows a higher score in confidence for those who completed the test than those who failed to do so although no significance was found (rank sum test p = 0.09, effect size $\eta ^2=0.102$).

In terms of the confidence in reading the result (Q12e), as in Fig. 10, 191 of 264 (72.3%) gave the highest score 5, 45 of 264 (17.1%) scored 4, and 17 of 264 (6.4%) scored only 1. We further examined what type of tests the users were less confident to read for those 73 users who scored under 5, and the results are given in Table 8. It is noted that the highest number 18 of 73 (24.7%) was from ‘Invalid’, but there are 32 of 73 (43.8%) from the combination of T1 and T5 in those scored less than 5. The results suggest that for the tests T1 and T5, the participants felt less confident in reading compared to the rest, which concord with the findings pertaining to the accuracy of reading test results (in Table 7).

**Table 8:** The counts for participants’ confidence levels scored under 5 when reading the result (Q12e) and the ground truth provided in their printed cards.

Q12e	Printed cards’ results					
	Positive (T1)	Strong positive (T2)	Weak positive (T5)	Negative (T3)	Invalid (T4)	Total
Scores < 5	15	8	17	15	18	73

### Feedback

The areas for future improvement have been identified from the user feedback. There were 123 free text comments received from the participants, which can be summarised in several aspects related to difficulty in using the test kit, instructions, risks/safety warnings, and UX study design. A total of 36 out of 123 (28%) users found the blood collector was difficult to use, such as the issues related to blood sample collecting included the blood bubbled into the test, difficult to expel blood, or unsure of the size of drop of blood required. Similar issues have been reported in the previous car-based study^26^. Regarding the instructions, 9 of 123 (7.3%) found it helpful to watch the video before the test, three users pointed out the layout of the instruction booklet was not easy to read, and two users raised the issue of hygiene and suggested the instruction should advise washing hands after the test.

Some users were concerned by the dexterity required to complete the test as they found it was difficult due to either shaky hands (two users), poor eyesight (one user), or disability (one user). One younger user was concerned that the task could be difficult for the elderly in terms of comprehending a large amount of information and uploading photos via mobile device. Therefore, extra help and consideration will be needed for those vulnerable people.

In terms of UX study design, six users found the amount of information required to read and the paperwork to process was overwhelming and off-putting. Three users mentioned that they were unclear about the purpose of using the printed test cards. Four users reported that they had a technical issue when uploading images using their mobile devices and had to use a laptop or send photos via their email. Six users suggested that plaster and tissue should be provided in the kit for the users. Four users mentioned the package received via postal were damaged, which suggests that a better package may be needed if using the postal service to deliver the test kits in the future.

## Discussion

This paper presents a home-based UX study for SARS-CoV-2 antibody rapid test kit via simulated LFIA testing. A summary of UX analysis methods used in this study and two related studies^23,26^ is provided in Table 9, which highlights the contribution of this study to the literature. Although containing a relatively smaller sample size than the other two studies, this study carried out a more in-depth analysis that covered the multidimensional aspects of UX analysis in terms of ease of use, effectiveness, efficiency, accuracy and satisfaction.

**Table 9:** Comparison of UX analysis methods used in this study and two related studies for LFIA self-testing for COVID-19.

	Study-1^23^	Study-2^26^	This study
**Environment**	At home	In car	At home
**Sample size**	N1: 10600; N2: 3800	1544	264
**Number of questions**	17	28	41
**Ease of use**	4 10-point Likert ratings	28 5-point Likert ratings (analysis for age & education groups)	25 polar questions
**Effectiveness**			
Completion rate (with valid C-line)	N1: 91.5%; N2: 94.4%	Not recorded	96.7%
Identify reasons for failure	Yes	Not recorded	Yes
Identify areas of difficulty	Yes	Yes	Yes
**Efficiency**			
Use of physical resource	Not recorded	Yes	Yes
Frequency to consult the instructions	Not recorded	Yes (analysis for age groups)	Yes (analysis for age groups)
Use of instruction video	Not recorded	Yes	Yes
Human support	Yes	Not recorded	Yes (analysis for age groups)
Mental workload	Not recorded	Not recorded	Yes (analysis for age & education groups)
**Accuracy (Kappa score)**	N1: 0.72, N2: 0.89 (real test results)	0.75 (real test results)	0.67 (simulated test results)
**Satisfaction (acceptability)**	2 polar questions	Not recorded	5 7-point Likert ratings

1. For ease of use, study-1^23^ used 4 10-point Likert ratings to check the user’s understanding of the instruction and ability to perform the test, this study contained 25 of 30 polar questions that measured the UX of a particular aspect of the testing kit (as seen in Fig. 6). The design of questions simplified the users’ tasks by replacing the 5-point Likert ratings used the study-2^26^ by the polar questions. A similar average UX score (95.27%) was obtained as in study-2^26^ (96.03%), which suggests the good user experience of the test kit for both using the AbC-19 LFIA test kit in cars and at home.
2. In terms of effectiveness, similar as highlighted in study-1^23^, the common difficult areas for completing the test were applying the blood drop to the test and collecting the blood using the blood collector (as in Fig. 7), which suggests these areas should be the focus for design improvement in the future.
3. In terms of efficiency, this study assessed the use of the physical resource, number of times to consult the instruction, use of instruction videos, human support, and mental workload, together with cross-examination for age and education groups. The results suggest the usefulness of the instruction video and recommend that additional support is needed for two age groups (< 18 and 60+) during the self-administrated test. NASA’s TLX was applied to assess the users’ perceived cognitive demand of the tasks, which may potentially help to reduce error and improve customer acceptance. As seen in Fig. 9, the participants scored the test highest for mental demand (31.5 ± 19.7) and lowest for physical demand (23.3 ± 17.2), the overall workload TLX score is 26.5 ± 19.1. A paper conducted a meta-analytic review^49^ about reference values and subscale patterns for TLX based on 556 studies across 18 domains, 4 technology areas and 6 global regions. All papers selected in this review were based on raw TLX including six subscales, the values for six subscales were rescaled to the 0–100 range and TLX was calculated as the mean of the subscales, which was the same approach used in our study. According to this review paper^49^, the average TLX score when used for the domain of manual labour (physical work) is 56 ± 12, and the average TLX when using handheld devices is 35 ± 16. In comparison, the TLX score (26.5 ± 19.1) achieved for the LFIA rapid test kit in this study is lower than these two aforementioned TLX scores for the domain of manual labour and when using handheld devices, which suggests that the general public have a relatively low level of mental workload when using LFIA self-testing at home.
4. For assessment of accuracy, substantial agreement was found via Kappa scores in all studies. In two related studies, the participants’ reading for actual LFIA testing results were compared to the clinicians’ or researchers’ results. Simulated test results were applied in this study, in which a controlled variation of T-line and C-line helped us to assess how participants performed when reading different types of test results. The results suggest that the categories of positive (T1) and weak positive (T5) were more difficult to interpret due to the relatively low T-line intensity when compared to the strong positive (T2) cases.
5. The assessment of satisfaction suggests that the end-users felt less confident in reading tests of T1 (positive), T5 (weak positive), and T4 (invalid) compared to the rest. However, overall the users felt the test kit was easier to use than they expected and a majority (87%) of users scored 5 over 7 and considered that the capability of the test kit met their requirements.

The findings from using simulated test results may also suggest the potential need for further investigation of the quantitative analysis of different COVID-19 antibody levels. Although there are studies that have reported the dynamic changes of SARS-CoV-2 antibody response^30,31,62^, there is a lack of investigation of the relationship between the T-line intensity and SARS-CoV-2 antibody levels in the literature. Some researchers^30^ are concerned that the reduction in SARS-CoV-2 IgG and neutralising antibody levels in the early recovery phase might have implications for immunity strategy. Another study^31^ also suggests further studies will be needed to define a quantitative protection threshold and rate of decline of antiviral antibodies beyond 90 days. Future work to investigate the relationship between the T-line intensity and SARS-CoV-2 antibody levels may provide new insight to fill these gaps in the literature.

Furthermore, the methodology and findings in this study may also provide values to other types of LFIA applications, not just COVID-19. For example, a better understanding of the area of difficulties in self-testing may help to improve the LFIA testing performance at home in general, also the approach for cross-examination of groups (age/education) helps to uncover the potential issues in different groups of the end-users so the additional support can be provided for those in need, such as the users who are younger than 18 and over 60.

Limitations of this study include the following aspects: (1) The questionnaire used in this study was based on modification and extension of our previous study for UX study of LFIA testing COVID-19 in cars^26^, which was not a validated questionnaire but was specifically designed for our study. Since we found there was a lack of well-defined measurement metrics for UX related to LFIA self-testing. Those related studies^17,19,22,23^ mainly focused on the assessment of accuracy in interpreting test results and tried to identify specific issues for their study based on the feedback received from their questionnaires. The questions in the industry standard benchmark for usability measures like SUS^63^ or USE^64^ were unsuitable for our study, such as the SUS question “I think that I would like to use this system frequently”, and the USE questions “It helps me be more effective”, “It helps me be more productive”, which are not applicable to the LFIA testing for COVID-19. Therefore, we applied self-defined measurements for UX analysis together with NASA-TLX for assessment of mental workload to explore the multiple aspects of user experiences. Modification of SUS questions as suggested in other study^65^ can be considered in the future study. (2) The assessment of mental workload based on the measure of TLX may be limited via only comparing to the reference value recommended in the literature^49^, rather than proposing criteria for target NASA-TLX for the home-based LFIA testing, which is beyond the scope of this study but will be considered in the future work. (3) Compared to related studies^23,26^, this study has relatively small samples. Also due to the restraints of responses to recruitment, some groups were over-represented (females) while other groups were under-represented (males). In terms of age distribution, according to NI 2011 Census^29^, 13.18% of the NI population (1.8 million) are aged 8-17 and 18.73% are aged over 60. For this study, we have 30 of 264 (11.3%) from under 18 and 48 of 264 (18.2%) from the group of age over 60, which was broadly similar to the NI population profile, but more volunteers with primary or secondary education will need to be included to improve the analysis related to the education groups.

## Conclusion

This paper presents a UX study to assess a home-based rapid LFIA test kit for SARS-CoV-2 antibody testing based on 264 participants in Northern Ireland. Overall, the users found the test kit easy to use and the areas of difficulty in completing the self-test were identified. The efficiency in terms of use of the physical resource, human support and mental workload was assessed. The statistical analysis found substantial agreement (Kappa score 0.67) between the test results that were interpreted by participants and the ground truth, although the users found the weak positives (with the faint test lines) difficult to read. The overall user feedback provided valuable information for possible improvement for the design of SARS-CoV-2 antibody testing kits and inform protocols for future UX studies.

## Supplementary Information

Supplementary Information.
