# Supplementary material for: User experience of home-based AbC-19 SARS-CoV-2 antibody rapid lateral flow immunoassay test
Source: Sci Rep. 2022 Jan 21;12:1173. doi: 10.1038/s41598-022-05097-y (PMC8782985; doi:10.1038/s41598-022-05097-y)
Supplement: Supplementary file 1 — Supplementary Information. [file 41598_2022_5097_MOESM1_ESM.pdf]

Supplementary Files: Tables, Questionnaire & Test Kit Instructions

User experience of home-based AbC-19 SARS-CoV-2 antibody rapid lateral flow immunoassay test

Min Jing[1], Raymond Bond[2], Louise J. Robertson[3], Julie Moore[3], Amanda Kowalczyk[3], Ruth Price[3], William Burns[1], M. Andrew Nesbit[3], James McLaughlin[1*], and Tara Moore[3,4*]

[1]Nanotechnology and Integrated Bioengineering Centre (NIBEC), School of Engineering, Ulster University, Jordanstown, UK
[2]School of Computing, Ulster University, Jordanstown, UK
[3]Biomedical Sciences Research Institute, Ulster University, Coleraine, UK
[4]Avellino USA, 1505 Adams Drive, Menlo Park, CA 94025, USA
[*]Joint corresponding authors: Prof. Tara Moore tara.moore@ulster.ac.uk, Prof. James McLaughlin jad.mclaughlin@ulster.ac.uk

Supplementary Table S1:

**Age Groups**	**7-17**	**18-30**	**31-60**	**60+**
**7-17**	1	-	-	-
**18-30**	0.46 (0.10)	1	-	-
**31-60**	0.50 (0.10)	0.73 (0.02)	1	-
**60+**	0.79 (0.03)	0.57 (0.10)	0.67 (0.03)	1

Table S1. Pair-wised Chi-square test p-values and effect size (in bracket) for users who consulted instruction 1-3 times in four age groups.

Supplementary Table S2:

**Age Groups**	**7-17**	**18-30**	**31-60**	**60+**
**7-17**	1	-	-	-
**18-30**	< 0.001 (0.632)	1	-	-
**31-60**	< 0.001 (0.602)	0.973 (0.003)	1	-
**60+**	< 0.001 (0.466)	0.100 (0.191)	0.003 (0.207)	1

Table S2. Pair-wised Chi-square test p-values and effect size (in bracket) for users received help from others in four age groups.

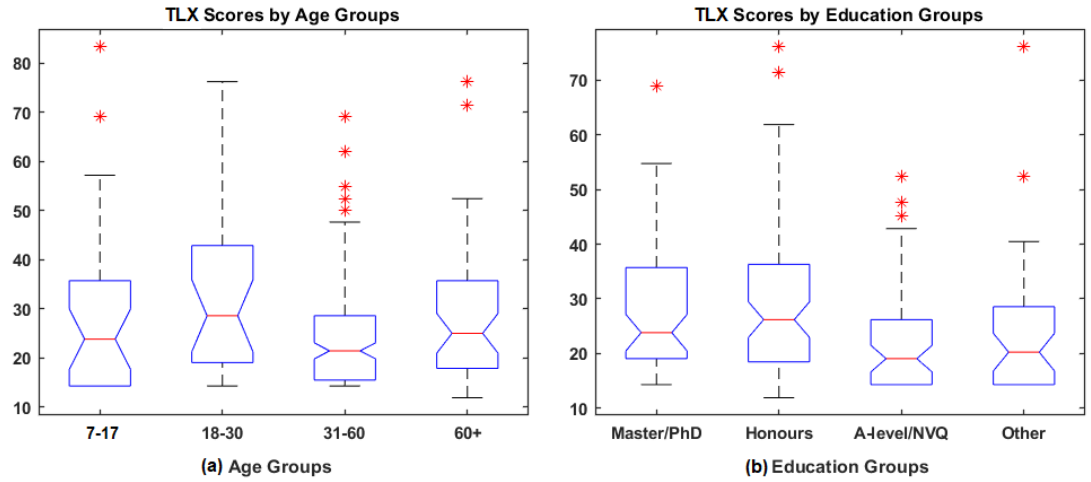

Figure S1. Boxplot for TLX scores in: (a) four age groups and (b) four education groups. (In the boxplot, the central lineindicates the median, whilst the bottom and top lines represent the 25th and 75th percentiles, respectively, with outliers plottedas an asterisk)

Home user feedback Survey

Many thanks for being willing to help us with our research.

We have now posted your test kit to you. When you receive the test please follow the instructions below. We ask you do this within 5 days of receiving the test.

Instructions

a) Make sure you have your mobile phone with you to take a picture of your test when complete.
b) Undertake the test as per the instruction leaflet

Please note for the purposes of this study the test provided within the kit is a "dummy kit" and will only show a control line. The 'dummy test' is unable to detect any antibodies related to the COVID-19 disease. Consequently no information regarding your COVID-19 antibody status will be obtained during the completion of this trial.

c) Complete the survey telling us what you think of the test!

Once you complete the test we'll email you your Amazon voucher to say thank you for your time and feedback.

If you have any questions please do not hesitate to contact our research team at pandemicstudy@ulster.ac.uk or Professor Tara Moore on 02870124577

Thank you!

This Questionnaire has been compiled to establish your personal views and user experience of the rapid home test for detection of antibodies to the SARS-CoV-2 virus. Please complete the details below and select your level of satisfaction for each question.

Please respond to the following statements by the marking yes or no for the appropriate answer.

Email address ______________________

Date of birth ______________________

Surname ______________________

Instruction video

Section Q 1: Outer Packaging

Q1a: Did the packaging provide sufficient protection to the kit materials __
Q1b: Were the kit materials easily accessed ______
Q1c: Did the packaging provide clear information to the type of test and materials inside _

Section Q 2: Collection of Finger Prick Blood Sample

Q2a: The lancet was easily identified ___
Q2b: The lancet cap was easy to remove __
Q2c: The fingerpick puncture was easy to perform __
Q2d: The correct amount of blood was collected from the fingerpick puncture ___
Q2e: The second lancet was required to be used _

Section Q 3: Application of Sample to Test

Q3a: The test was easily identified _
Q3b: The test was easy to remove from the foil packaging __
Q3c: The correct place to apply the sample ('sample hole') was easily identified _
Q3d: The blood was easily expelled from the blood collector to the test __

Section Q 4: Application of Test Solution to Lateral Flow device

Q4a: The test solution was easily identified _______
Q4b: The twist cap was easy to remove _
Q4c: The test solution was easily applied to the sample hole on the device ______
Q4d: There was no test solution left in the container _____

Section Q 5: Development of a control line

A control line (C-line) was present within the test window ◯ Yes ◯ No

Please take a picture of your test and upload here

Section Q 6: Instructions for Use

Q6a: The instructions provided were easy to follow _____
Q6b: The instruction video was watched prior to performing the test ____
Q6c: The user steps were simple and easy to perform _____
Q6d:The items in the kit were appropriately labelled _____
Q6e: The test is in an easy to use format _____
Q6f: How many times during the procedure did you need to consult the instructions 1-3☐ 4-6☐ 6- 9☐ 10+☐

Section Q 7: Risks and Warnings

Q7a: The lancet was understood to contain a needle _____
Q7b: The risks associated with the lancet were clearly understood _____
Q7c: The potential for small components to be a choking hazard was clearly understood _____
Q7d: The correct way to dispose of the kit following use was understood _______
Q7e: If unused the second lancet was clicked prior to disposal _______

Section Q 8: Reason for test failures

Q8a: Was the test completed and the control line obtained? ◯ Yes ◯ No

Q8b:

Reason for failure

- ☐ Could not use the Lancet
- ☐ Could not get a good sized drop of blood to form
- ☐ Could not collect the blood using the blood collector
- ☐ Could not add the blood to test
- ☐ Could not open test solution
- ☐ Could not add the test solution to the test
- ☐ Other

(Please select all that apply)

Q8c: Please provide details on reasons for failure ______________________________

Section Q 9: Difficulties that did NOT lead to a test failure

Q9a: During the completion of the test, please identify anyareas of difficulty by ticking the most appropriate box below (please do not include any area that lead to a failure as marked in Section Q8).
If you experienced no difficulties please select the first box.

- ☐ No Difficulties
- ☐ Difficulty using the Lancet
- ☐ Difficulty in getting a good sized drop of blood to form
- ☐ Difficulty in collecting the blood using the blood collector
- ☐ Difficulty adding the blood sample to the test
- ☐ Difficulty in opening the test solution
- ☐ Difficulty adding the test solution to test
- ☐ Other

Q9b: Please include reasons not detailed above ______________________________

Q9c: Did you receive help from another person throughoutthe study? ◯ Yes ◯ No

Q9d: Please describe the type of help received ______________________________

Section Q 10: Interpretation of test result provided on printed card

Please remove the test card provided within the kit and complete the following questions.

Q10a: Please identify the test number on the printed card provided within the pack:

- ◯ 1
- ◯ 2
- ◯ 3
- ◯ 4
- ◯ 5

Q10b: The result of the test provided on the printed cards was:

- ◯ POSITIVE
- ◯ NEGATIVE
- ◯ FAILED / INVALID
- ◯ NOT SURE

Please take a picture of the test printed on the card and upload here:

Q10c: The results of the printed test provided on the cardwas easily interpreted based on the information provided in the instructions

◯ Yes ◯ No

Section Q 11: Task Load Index (TLX)

Q11a: How mentally demanding was the task? ______________________ Low 1 ☐ 2 ☐ 3☐ 4 ☐ 5 ☐ 6☐ 7☐ High
Q11b: How physically demanding was the task? ______________________ Low 1 ☐ 2 ☐ 3☐ 4 ☐ 5 ☐ 6☐ 7☐ High
Q11c: How hurried or rushed was the pace of the task? ________________ Low 1 ☐ 2 ☐ 3☐ 4 ☐ 5 ☐ 6☐ 7☐ High
Q11d: How successful were you in accomplishing what you were asked to do?__ Good_1 ☐ 2 ☐ 3☐ 4 ☐ 5 ☐ 6☐ 7☐ Poor
Q11e: How hard did you have to work to complete the task and get a result?____ Low1 ☐ 2 ☐ 3☐ 4 ☐ 5 ☐ 6☐ 7☐ High
Q11f: How insecure, discouraged, irritated, stressed, and annoyed were you? ___ _Low 1 ☐ 2 ☐ 3☐ 4 ☐ 5 ☐ 6☐ 7☐ High
______

Section Q 12: Comfort and Acceptability

Q12a: If I needed a testing kit, the capabilities of this kit would meet my requirements: _1 ☐ 2 ☐ 3☐ 4 ☐ 5 ☐ 6☐ 7☐
Q12b: The testing kit was easy to use __ 1 ☐ 2 ☐ 3☐ 4 ☐ 5 ☐ 6☐ 7☐
Q12c: How easy to use did you expect the testing kit to be? ________________ 1 ☐ 2 ☐ 3☐ 4 ☐ 5 ☐ 6☐ 7☐
Q12d: How confident were you in using the kit? ______________________1 ☐ 2 ☐ 3☐ 4 ☐ 5 ☐ 6☐ 7☐
Q12e: How confident were you in reading the result? __________________1 ☐ 2 ☐ 3☐ 4 ☐ 5 ☐ 6☐ 7☐

Section 13: Additional feedback

If there is any additional feedback not covered above that you would like to provide regarding your experience when performing the AbC-19TM rapid test please include below; ______________________________

29/07/2020 15:31

UK-RTC

AbC-19™ RAPID TEST

Instructions

1. Intended Use

The AbC-19™ Rapid Test is a single – use test for the detection of IgG antibodies in human capillary whole blood.

When the body is invaded by harmful bacteria or viruses, the immune system responds by producing disease specific antibodies. These antibodies help fight the infection and in some instances provide protection against future infections (immunity).

Using a blood sample from a finger-stick puncture the AbC-19™ Rapid Test will identify the presence of antibodies produced in response to the SARS-CoV-2 virus (the virus responsible for the COVID-19 disease), signifying a recent or previous infection by the virus.

2. Intended End User

The AbC-19™ Rapid Test is intended to be used by healthcare professionals.

3. Background

The SARS-CoV-2 virus is a member of the Coronavirus family (CoV). In humans this virus family is capable of causing illnesses that range from the common cold, to more serve conditions such as serve acute respiratory syndrome (SARS) and the COVID-19 disease.

Symptoms of COVID-19 can vary but most commonly include a fever, tiredness, dry cough, shortness of breath, loss of taste and smell and difficulties breathing. Some patients are asymptomatic and show no symptoms.

Antigen and antibody tests are currently the two main types of tests being used to test for the COVID-19 disease. Antigen tests are able to detect the presence of the virus and confirm whether a patient is currently infected. In contrast, an antibody test does not identify the virus itself but measures the body's immune response to the invading virus, by detecting the presence of the disease specific antibodies.

The immune response typically involves an initial production of short-lived Immunoglobulin M (IgM) antibodies, followed by a second response and the production of Immunoglobulin G (IgG) antibodies and in some people Immunoglobulin A (IgA).

The AbC-19™ Rapid Test detects IgG antibodies. Current evidence suggests these antibodies become detectable sometime between 4-19 days after the onset of symptoms, which is why the AbC-19™ Rapid Test should be performed **after day 14**. It is not known how long IgG antibodies for SARS-CoV-2 are present in the blood, but they typically persist for several months.

4. Limitations

AbC-19™ Rapid Test has been validated for use with blood samples obtained from a finger- stick puncture. **No other sample types should be used.**

AbC-19™ Rapid Test indicates the presence of SARS-CoV-2 IgG antibodies and should not be used as the sole criteria for the confirmation /exclusion of SARS-CoV-2 infection or confirmation of immunity against COVID-19.

AbC-19™ Rapid Test may give a negative result for the presence of SARS-CoV-2 antibodies (IgG), if the test is performed less than 14 days after the first signs of infection. IgG antibodies may be present but below the detection limit of the test. Other contributing factors towards a false negative include a weakened immune system.

5. Disclaimer

The manufacturer of this product shall not be liable for any losses, liability, claims, costs or damages whether direct or indirect or consequential arising out of or related to an incorrect test result, whether positive or negative, as indicated by this product.

Do not make any medical or personal safety decisions based on the results of this test without consulting your doctor first.

Symbol Key

IVD *In vitro* diagnostic medical device

LOT Batch code

REF Catalogue number

Contains sufficient for <n> tests

Consult the instructions for use

Temperature limit

Manufacturer

Date of manufacturer

Do not re-use

Use-by-date

Keep out of sunlight

Do not use if packaging is damaged.

CE mark

Instructional Video

For additional guidance on how to perform the AbC-19™ Rapid Test please watch our short instruction video.

To access the video scan the QR code below or visit:

bit.ly/AbC-19Video

6. Warnings and Precautions

Please read the instructions provided carefully before performing the test. Failure to follow the test procedure could lead to inaccurate results.

The AbC-19™ Rapid Test is a single use *in vitro* diagnostic (for use outside of the body) test which cannot be re-used.

Blood samples must not be taken from fingers on the side affected by a mastectomy.

It may not be possible to obtain a blood sample using a finger-stick puncture if suffering with poor peripheral circulation (e.g. suffering with peripheral edema).

Do not use the lancet prior to performing the test. The lancets are of single use only.

The lancet contains a needle, please keep out of reach of anybody under the age of 16 and pets. If any of the kit materials are swallowed, seek medical advice immediately.

Do not use a single puncture site more than once, this can lead to bacterial contamination and infection.

Handle all parts of the kit, both during and after use, as potential infectious material, taking necessary blood contact precautions.

This test is not suitable for use by anyone suffering with a blood coagulation disorder or under the age of 8.

Children between 8 and 16 should not perform the test without adult supervision.

The kit materials are not considered dangerous according to the 2012/18/EU and 1272/2008 Directives.

Whilst every effort has been taken to ensure the accuracy of this product, as the product is used beyond the direct control of the manufacturer the result may be affected by environmental factors and/or user error should the instructions not be followed.

7. Storage and Handling

Store the AbC-19™ Rapid Test kits in a cool, dry place between 5-30°C, away from direct sunlight. Do not store on or above a radiator.

Do not touch the test with wet hands. Dry hands thoroughly prior to taking the blood sample.

Do not remove the test from its packaging until ready to perform. Once the test has been removed please perform the test immediately.

The test should be performed at **room temperature** (15-25°C).

Do not use the AbC-19™ Rapid Test if the box or kit contents are damaged.

8. Test Principle

Only a small amount of blood is required to perform the test. Using the provided lancet a small blood sample is obtained from a finger-stick puncture and collected via the provided blood collector.

The test is performed by applying the collected blood to the sample hole, followed by the application of the provided test solution. Once applied this mixture is absorbed by the paper strip and will begin traveling down from the sample hole and across the viewing window.

If SARS-CoV-2 antibodies are present within the blood sample these disease specific antibodies will attach themselves to the first line, the test line (T-line) resulting in the formation of a visible red line. In the absence of SARS-CoV-2 antibodies, no attachment will occur at the T line, resulting in no visible line at the T-line position.

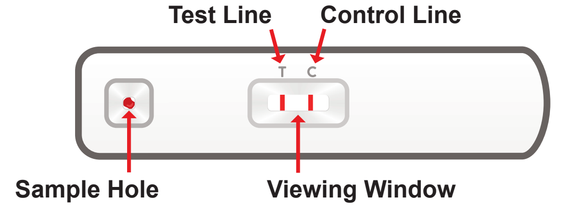

The second line to appear is an internal control line (C-line). This line will only appear if the test procedure is followed correctly.

9. Interpretation of Results

Once the test has been performed up to two lines can appear on the test.

The line furthest away from the sample hole is the control line (C-line). The C- line is always present if the test has been performed correctly. The C-line must be present when reading the results. In the absence of a C-line the test is invalid and the result must not be used. The test will need to be repeated using a new test device and fresh blood sample.

The presence of only a C-line indicates a **NEGATIVE** result.

The line closest to the sample hole, the test line (T-line), will only be visible if you have SARS-CoV-2 IgG antibodies present within the blood sample. The presence of a T-line alongside a C-line is a **POSITIVE** result, indicative of a recent or previous SARS-CoV-2 infection.

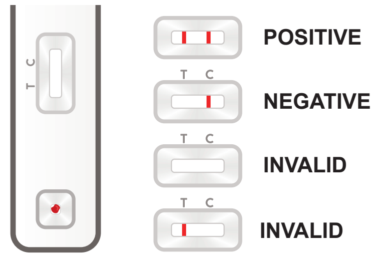

The absence of any lines or the presence of the T-line alone indicates the test has not been performed correctly. The results of the test are invalid and must not be used. The test will need to be repeated using a new test and fresh blood sample.

10. Performance Characteristics

As detailed in the table below a known population of 450 negative samples were tested alongside 203 known positive samples. The positive population of 203 samples were obtained from donors at least 14 days after the onset of COVID-19 symptoms and were verified as positive using a commercial IgG SARS-COV-2 ELISA kit.

		AbC-19™ Rapid Test	
		Positive	Negative
ELISA	Positive	199	4
	Negative	2^	448*

* 98 pre-pandemic negative samples not tested by ELISA

^ 2 pre-pandemic negative samples not tested by ELISA

Clinical Sensitivity and Specificity is as follows:

Sensitivity: 98.03% (95% CI[a]: 95.03% - 99.46%)

Specificity: 99.56% (95% CI[a]: 98.40% - 99.95%)

[a] = Confidence Interval

Cross reactivity:

Known positive serum samples from other viral infections were tested as follows (value in square brackets refers to the number tested) **Seasonal Coronavirus** (HCoV-NL63 [x5] and HCoV-229E [x5]), Influenza A [x5], H5N1 Influenza [x1], influenza B [x6] Respiratory Syncytial Virus (RSV) [x6], Haemophilus influenzae type b [x5] and Bordetella pertussis [x1]. No cross reactivity was observed, with all tests demonstrating a negative result AbC-19™ Rapid Test.

Interference:

A range of substances were tested using the AbC-19™ Rapid Test for positive and negative interference. No false positives or false negatives were recorded at the concentrations detailed in the table below;

Substance	Upper limit of normal serum levels mg/dL	Level Tested mg/dL
Unconjugated Bilirubin	2	40
Cholesterol (total)	<200	400
Triglyceride	200	1500
IgG	1400	4,200
IgM	250	750
Haemoglobin	17.5	1000
Biotin	0.117	0.351
Acetaminophen (paracetamol)	5.2	15.6
Acetylsalicylic acid (asprin)	1	3
Ibuprofen	7.3	22
Caffeine	3.6	11

References

Guarner J. Three emerging coronaviruses in two decades the story of SARS, MERS, and now COVID-19. Am J Clin Pathol. 2020. March; 153 (4): 420–5.

Huang C, Wang Y, Li X, et al. Clinical features of patients infected with 2019 novel coronavirus in Wuhan, China. Lancet. 2020. February; 395 (10223): 497–506.

Bénézit F, Le Turnier P, Declerck C et al. Utility of hyposmia and hypogeusia for the diagnosis of COVID-19. Lancet Infect Dis. 2020. April; S1473-3099 (20): 30297-8

Chaplin DD. Overview of the Immune Response, J Allergy Clin Immunol. 2010. Feb; 125 (2 Suppl 2): S3–23.

Clem AS. Fundamentals of Vaccine Immunology. J Glob Infect Dis. 2011. Jan-Mar; 3 (1): 73–78.

Long Q, Liu B, Deng H, et al. Antibody responses to SARS-CoV-2 in patients with COVID-19. Nat Med. 2020. April; 26: 845–848

Hortensius J, Slingerland RJ, Kleefstra N, et al. Self-Monitoring of Blood Glucose: The Use of the First or the Second Drop of Blood. Diabetes Care. 2011. Mar; 34 (3): 556–560.

Procedures and devices for the collection of diagnostic capillary blood specimens; approved standard 6th edition. CLSI Standard, GP42- A6

WHO Guidelines on Drawing Blood: Best Practices in Phlebotomy. Geneva: World Health Organization; 2010. 7, Capillary sampling. Available from: https://www.ncbi.nlm.nih.gov/books/NBK138654/

Turn page for step-by-step instructions ›

Product Code: FG-FD51920, Document No: IFU163, Version: 2.00, Issue Date: September 2020 Abingdon Health, Sand Hutton, York, YO41 1LZ, United Kingdom, Tel: +44 (0) 1904 40 60 80

When to test?

The AbC-19™ Rapid Test should not be used until **at least 14 days** after the onset of symptoms.

Symptoms of COVID-19 are:

- **A high temperature**
- **A new continuous cough**
- **A loss or change to sense of smell or taste**

Before you start...

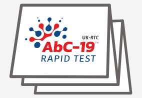

Read the step-by-step instructions several times until you are confident you understand each stage.

For additional guidance, watch our short instruction video.

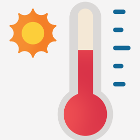

Ensure hands are warm.

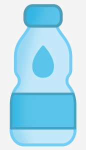

Ensure good hydration.

KIT MATERIALS

Materials Provided:

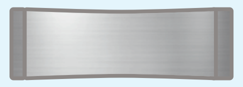

1x Test

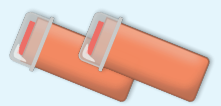

2x Single-Use Lancets

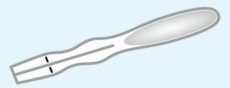

1x Blood Collector

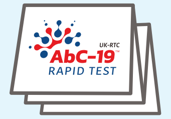

1x Instructions

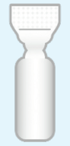

1x Test Solution

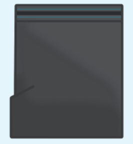

1x Waste Bag

Additional Materials Needed:

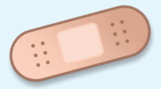

Plaster

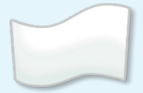

Tissue

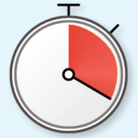

Timer

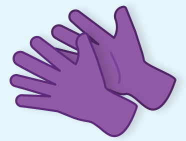

Medical Gloves

HELPFUL TIPS

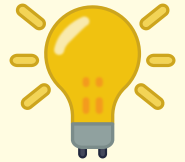

Perform the test at **room temperature** (15-25°C) in a well-lit area.

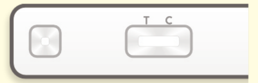

When performing the test, lie the test on a clean flat surface.

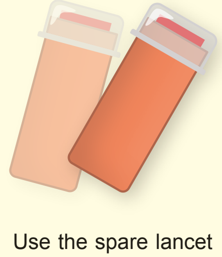

Use the spare lancet if you have problems getting enough blood for the sample.

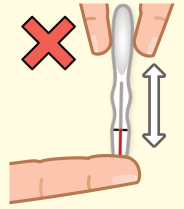

DO NOT hold the blood collector vertically when collecting the blood.

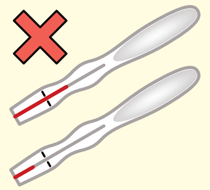

DO NOT over-fill or under-fill the blood collector.

STEP 1: PREPARE

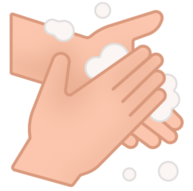

1. Prepare by washing hands with nothing but soap and **warm water**. Drying thoroughly. Do not apply any hand cream or hand sanitiser.

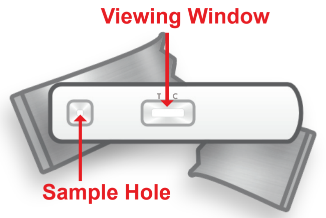

2. Open foil pouch and remove test. Discard silica gel packet.

STEP 2: SAMPLE

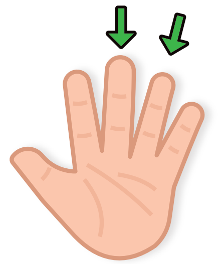

3. Blood should be collected from ring or middle finger of the non-dominant hand.

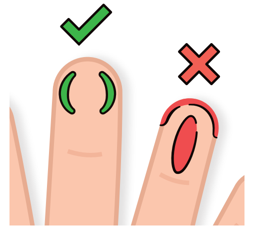

4. The finger stick puncture should be performed on the **side of the fingertip** (just off centre), marked by the green area.

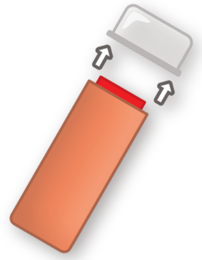

5. To perform the finger-stick puncture, first remove the protective cap from the lancet.

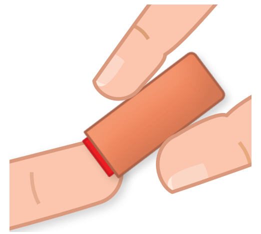

6. Place the **raised red platform** against the side of the fingertip. Apply gentle and steady pressure for 2-3 seconds until a click is heard.

STEP 3: COLLECT

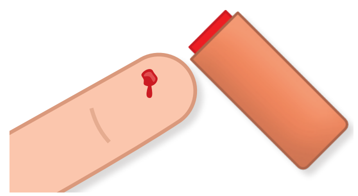

7. Wait a few seconds for a drop of blood to form. If a drop does not form very gently squeeze the sides of the finger.

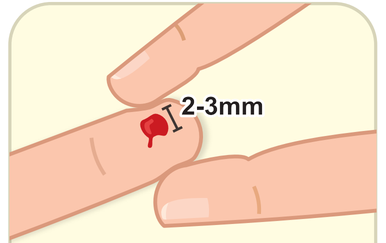

Ensure a good size drop of blood has formed before collecting the blood sample.

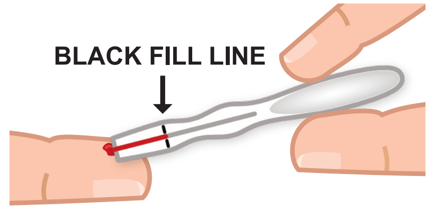

8. Holding the blood collector horizontally at a slight angle, gently touch the tip to the blood drop. The blood will automatically be drawn up the blood collector to the black fill line.

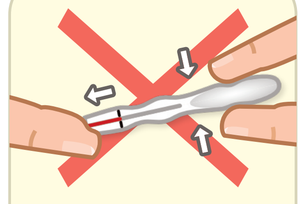

DO NOT squeeze the bulb or push the blood collector into the puncture site when collecting blood.

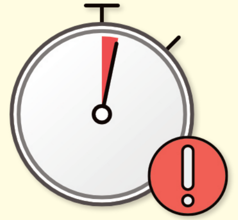

Once collected the blood will begin to clot, move to step 4 without delay.

STEP 4: RUN TEST

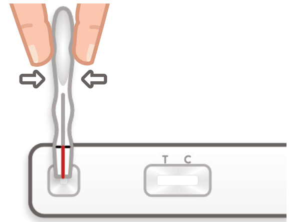

9. Holding the blood collector straight, gently touch the centre of the sample hole with the tip and squeeze the bulb carefully to add the blood to the test.

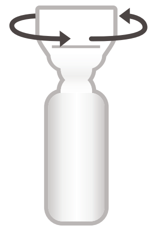

10. Twist and turn the top of the test solution to break the seal.

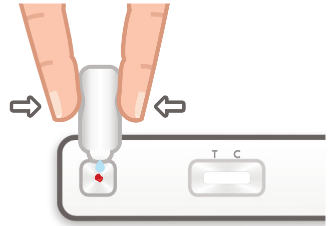

11. Apply the test solution to the sample hole on the test, one drop at a time, until there is no test solution remaining.

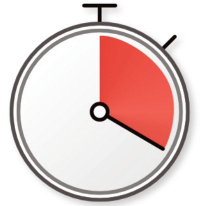

20 Minutes

12. Wait 20 minutes before reading the results.

STEP 5: RESULTS

Read the results immediately following the 20 minute wait time.
Reading too late can give inaccurate results.

13. After 20 minutes look at the viewing window to interpret your results. Your test will have...

Lines can only appear in the positions shown, but the colour intensity of the lines can vary.

2 Lines (T and C) = POSITIVE

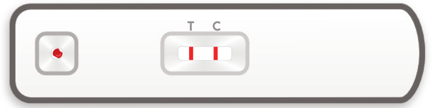

1 Line (C) = NEGATIVE

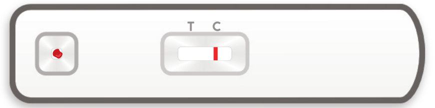

No Lines *or* 1 Line (T) = INVALID

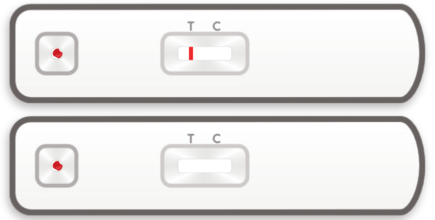

STEP 6: DISPOSAL

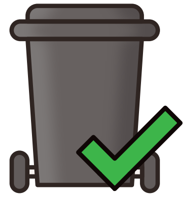

14. Place all kit materials in the waste bag, seal and place in the general waste. Press and click any unused lancets before disposing.

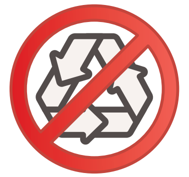

IMPORTANT:
The AbC-19™ Rapid Test is not suitable for recycling.

FURTHER ASSISTANCE

For additional assistance when performing and interpreting the AbC-19™ Rapid Test please contact our helpline:

+44 (0) 2890 394 258 (support lines are open 24 hours, seven days a week)

abc19support@mplcontact.com
